# Photochemical and Cloud and Aerosol Aqueous Contributions to Regionally-Emitted Shipping and Biogenic Non-Sea-Salt Sulfate Aerosol in Coastal California

**DOI:** 10.1021/acsestair.4c00352

**Published:** 2025-03-19

**Authors:** Nattamon Maneenoi, Lynn M. Russell, Sanghee Han, Jeramy L. Dedrick, Abigail S. Williams, Veronica Z. Berta, Christian Pelayo, Maria A. Zawadowicz, Arthur J. Sedlacek, Israel Silber, Mandy Thieman, David Painemal, Samuel S. P. Shen

**Affiliations:** † Scripps Institution of Oceanography, University of California, San Diego, La Jolla, California 92037, United States; ‡ Brookhaven National Laboratory, Upton, New York 11973, United States; § Pacific Northwest National Laboratory, Richland, Washington 99352, United States; ∥ NASA Langley Research Center, Hampton, Virginia 23681, United States; ⊥ San Diego State University, San Diego, California 92182, United States

**Keywords:** Aerosol, nonsea-salt (NSS) sulfate, marine biogenic emissions, shipping emissions, oxidation processes, photochemical reactions, cloud-aqueous reactions, atmospheric sulfate formation

## Abstract

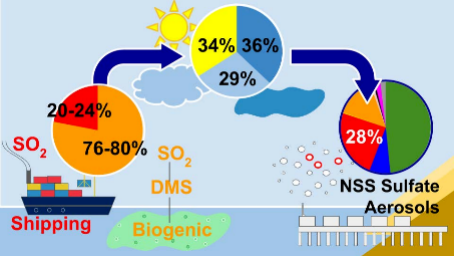

Aerosol nonsea-salt sulfate (NSS sulfate) forms in the atmosphere by secondary reactions of emissions from marine phytoplankton and shipping, with gas-phase as well as cloud and aerosol aqueous reactions controlling production. Twelve months of Atmospheric Radiation Measurements (ARM) during the Eastern Pacific Cloud Aerosol Precipitation Experiment (EPCAPE) at Scripps Pier in La Jolla, California, showed the highest NSS sulfate mass concentrations occurred for the northwesterly back-trajectories over 64% of the year, with an average of 0.90 μg/m^3^ that contributed 76% of annual NSS sulfate concentration. Multiple Linear Regression (MLR) and a refractory black carbon tracer method attributed 76–80% of the regionally emitted sulfur dioxide (SO_2_) sources of submicron NSS sulfate to marine biogenic emissions and 20–24% to shipping emissions. MLR for oxidation processes explained 21% of the variability with Downwelling Shortwave Radiation (DSW) driving photochemical reactions to account for 34% of annual regional sulfate production, Upwind Cloud Vertical Fraction (UCVF) controlling cloud-associated oxidation to account for 29%, and relative humidity (RH) describing aerosol-phase oxidation to account for 36%. NSS sulfate was correlated moderately to UCVF during April-June and August but to RH in October-January. These findings show the apportionment of SO_2_ emissions to biogenic and shipping sources and provide observational constraints for the mechanisms for sulfate production from SO_2_ in the atmosphere.

## Abstract 2

Aerosol sulfate in coastal regions has contributions from marine biogenic and shipping emissions, but atmospheric oxidation transforms the emitted gases to particles. The findings provide observational constraints on sulfate sources and cloud and aerosol processes, quantifying their impact on air quality and climate.

## 1 Introduction

Sulfate is a major component of ambient aerosols in coastal regions and globally, contributing 32–77% of submicron particle mass concentrations.^1^ Secondary sulfate aerosol from both anthropogenic and natural sources can influence the radiation budget of the atmosphere, though the extent of this effect remains uncertain due to the distribution and variability of aerosol sulfate sources and formation processes.^1−5^ Identifying the sources and processes affecting sulfate particle concentrations is crucial for quantifying regional climate effects and evaluating global models. Long-term observations provide the constraints needed to quantify these sources and processes contributing to sulfate production in different regions.

SO_2_ is the primary gaseous precursor of sulfate particles and is predominantly emitted by anthropogenic coal burning and other combustion of sulfur-containing fossil fuels.^6−11^ Shipping emissions constitute a significant source of SO_2_ emissions in coastal areas.^12−16^ A significant contribution to sulfate may come from marine biogenic sources, where marine phytoplankton produce dimethyl sulfide (DMS) that is transferred to the air and oxidized by reacting with atmospheric oxidants to produce SO_2_, Methanesulfonic Acid (MSA), and nonsea-salt sulfate.^17−20^ Previous studies have noted enhanced DMS levels during productive summer months, attributed to warmer temperatures and enhanced photochemical oxidation.^21−25^ DMS emission flux from the ocean is influenced by sea surface temperature (SST), wind speed, and DMS concentration in seawater.^21,22^ Another marine source of sulfate aerosols is sea salt; however, it accounts for a small fraction (<5%) of the sulfate mass concentration in fine particles (<1 μm).^24,26,27^ The sources and their contributions to the sulfate aerosol budget vary by location, meteorology, and season.^18,24,28−31^ The low contributions from coal burning in the western United States means that nonsea-salt sulfate (NSS sulfate) in California coastal regions is primarily emitted by marine shipping and biogenic emissions, but the apportionment between these sources has changed as ship fuel regulations have been updated^32,33^ and has not been characterized.

Sulfur dioxide (SO_2_) emissions are oxidized to sulfate during atmospheric transport, with the concentrations of atmospheric oxidants controlling sulfate formation.^16,34,35^ Specifically, sulfate can be formed through homogeneous reactions in the gas phase through oxidation of SO_2_ by OH and NO_3_ radicals.^1,36^ Sulfate in aerosol particles is also produced efficiently by heterogeneous or aqueous-phase reactions, in which ozone (O_3_) and hydrogen peroxide (H_2_O_2_) act as aqueous-phase oxidants. Aqueous production of sulfate has been identified as the main contributor to sulfate production on the global scale.^1,37,38^ Aqueous-phase pathways can occur in water-containing aerosol particles during high RH conditions or when SO_2_ is taken up in cloudwater.^35,39−45^ There are few observations of the contributions of aqueous-phase pathways to sulfate production in Southern California regions, and most results to date are limited to local effects rather than upwind cloud processing.^40,46,47^

Scripps Pier in La Jolla, California, frequently experiences air masses influenced by the shipping activities at the Ports of Los Angeles and Long Beach (LALB) and presents an excellent opportunity to examine the influence of mixed urban and coastal transport on aerosol properties in Southern California.^48−50^ Previous studies have investigated the composition of coastal aerosols at Scripps Pier and established that ambient aerosols in this region are primarily composed of organic components and sulfate, with sulfate mass fractions ranging from 37% to 47%.^49−51^ Elevated submicron mass concentrations have been attributed to primary emissions from sulfur-containing fuel sources from oceangoing ships.^48^ Previous filter-based triple oxygen isotope measurements of sulfate at the same sampling site in 2008 reported that 10–44% of nonsea-salt sulfate mass concentration below 1.5 μm were emitted by ships.^52^ However, challenges persist in comprehensively assessing NSS sulfate aerosol sources and associated atmospheric processes due to the short-term and intermittent sampling periods of earlier studies. As a result, there is still uncertainty regarding the degree to which various sources and meteorological conditions throughout the year impact the sulfate composition of ambient coastal particles.

This study uses year-long ambient observations from February 2023 through February 2024 at Scripps Pier as part of the Eastern Pacific Cloud Aerosol Precipitation Experiment (EPCAPE), a project of the U.S. Department of Energy Atmospheric Radiation Measurement (ARM). The study investigates the sources of SO_2_ contributing to NSS sulfate aerosol and the processes by which SO_2_ is oxidized in the Southern California coastal region. The focus is on the attribution of the NSS sulfate mass concentration associated with the most common airmass back-trajectory patterns. The results reveal the contributions of both regionally emitted marine biogenic and shipping emissions and show the relative importance of photochemical oxidation as well as two kinds of aqueous reaction pathways: cloudwater and aerosol water.

## 2 Methods

The Scripps Pier (32.87° N, 117.26° W) extends 300 m west of the shoreline, providing an ideal location to investigate the impact of coastal transport on aerosol properties in the Southern California region. The Aerosol Observing System (AOS) was deployed on Scripps Pier from 15 February 2023 to 14 February 2024.^53,54^ Meteorological conditions, such as ambient air temperature, relative humidity (RH), and precipitation, during the campaign were provided at 1 min time resolution by the ARM Surface Meteorology Systems (MET) using a Vaisala humidity and temperature probe (HMP155) mounted on the inlet of the AOS.^55^ In-situ measurements of sea surface temperature (SST) and Chlorophyll *a* (Chl *a*) concentration were taken at frequent intervals from surface seawater at the Scripps Pier, provided by the SCCOOS Automated Shore Station (SASS) Program.^56^

### 2.1 Online Aerosol Composition Measurements at Scripps Pier

The Aerodyne Aerosol Chemical Speciation Monitor (ACSM; Aerodyne Research Inc., Billerica, MA) was deployed in the AOS with aerosol inlet height of 18 m above mean sea level and provides 30 min averaged chemical composition of NR-PM_1_ (40 nm to 1 μm particle size), including nonrefractory (NR) organic components, sulfate, nitrate, ammonium and chloride. The ACSM NR aerosol composition is operationally defined as aerosol components that evaporate in the 600 °C vaporizer. Particles are expected to be trapped, volatilized rapidly, and detected on impact with the heated vaporizer, but some particles may instead rebound on impact with the vaporizer. To account for this rebound, a collection efficiency (CE) correction was applied to the ACSM measurements. In this study, the monthly CE values were determined based on the slopes fitted to the 23-h averaged NR mass from the ACSM and the integrated particle number size distribution-derived NR mass concentration for each month.^57,58^ The 23-h averaged NR mass concentration was estimated from the integrated particle number size distribution, with refractory black carbon (rBC), sea salt, and dust mass concentrations subtracted.^57,58^ Further details of the general ACSM setup and operation in the AOS can be found in Watson et al.^59^ and Theisen et al.^54^

A Single Particle Soot Photometer (SP2) was also deployed in the AOS and utilizes scattering and incandescence signals produced by black carbon-containing particles (50–500 nm diameter) to provide measurements of the refractory black carbon (rBC) mass concentration every 1 min.^60^

Online measurements were averaged to 3-h intervals to correspond with the 3-hourly back trajectories described in Section 2.3.

### 2.2 Filter Sample Collection and X-ray Fluorescence (XRF)

Filter samples were collected daily from 19:00 each evening to 18:00 the following day (local time, PST/PDT) using an inlet situated approximately 14 m above mean sea level on 37 mm Teflon filters (Pall Corp., Ann Arbor, MI). These filters were positioned downstream of a 1-μm sharp-cut cyclone (SCC 2.229 PM1, BGI Inc., Waltham, MA) within the AOS container on the Scripps Pier. Each filter set maintained a flow rate of approximately 10 LPM using a single flow meter and flow controller (Alicat Scientific, Tucson, AZ). Samples were transported back to the laboratory and stored frozen in airtight bags.^49,61^ A subset of 104 filters collected on Wednesdays and Saturdays each week were sent to Chester Labnet (Tigard, Oregon) for XRF analysis providing concentrations of elements, including Na, Mg, Al, Si, P, S, Cl, K, Ca, Ti, V, Cr, Mn, Fe, Co, Ni, Cu, Zn, Br, Rb, Sr, Zr, Ag, Pb, and Ba.^61−63^

The mass concentrations of sea salt, sea salt sulfate (SS sulfate), sea salt chloride (SS chloride), and dust were calculated from XRF-measured elemental concentrations. Sea salt particle mass concentrations were determined as XRF Na (μg/m^3^)*1.47 + XRF Cl (μg/m^3^).^64−67^ The SS sulfate concentrations were estimated by multiplying the ratio of sulfate (μg/m^3^) to XRF Na (μg/m^3^) in seawater, which is constant at 0.25 across major water masses in the ocean.^24,46,68^ The NR NSS Cl concentrations were calculated as NR Cl - (XRF Na*(Molecular weight of Cl/Molecular weight of Na)*CE_Sea salt_) (μg/m^3^), where CE_Sea salt_ ∼ 0.02.^69^ The mass of dust was calculated from XRF metal concentrations, assuming dust consists of MgCO_3_, Al_2_O_3_, SiO_2_, K_2_O, CaCO_3_, TiO_2_, Fe_2_O_3_, MnO, and BaO after excluding the mass associated with sea salt.^27,70^

The sea salt, SS sulfate, and dust components had mass above the detection limit (3 times the uncertainty) for 91% of the samples. The weekly averaged mass concentrations of sea salt, SS sulfate, and dust were calculated as the arithmetic mean of three filters analyzed by XRF for each week. NSS sulfate and chloride concentrations were calculated by subtracting the weekly average SS sulfate and chloride values from the respective 3-hourly sulfate and chloride mass concentrations measured by the ACSM.^58^

### 2.3 Back-Trajectories and Transport Clustering

The surface air mass trajectories (ARMTRAJSFC) used in this study^71^ were 48-h ensemble mean surface back trajectories calculated every 3 h (UTC) and originating at Scripps Pier at 0–50 m above mean sea level using the Hybrid Single-Particle Lagrangian Integrated Trajectory (HYSPLIT) model with the fifth generation European Centre for Medium-Range Weather Forecasts (ECMWF) atmospheric reanalysis^72^ at 0.25 degree and 1-h spatial and temporal resolutions. The trajectories were clustered using a k-means clustering algorithm to obtain prototypical trajectory paths during the campaign to represent the origin and travel path of air masses^50,73^ (Figure S1).

### 2.4 Retrieved Regional Ship Count, SST, and Chlorophyll *a* Concentration

Real-time tracking and monitoring of regional ships and vessel traffic were downloaded from the NOAA Automatic Identification System (AIS) (currently available through 2023). The hourly gridded ship point density was processed including passenger, cargo, and tanker vessel types and covering from latitude 32° to 34.5° N and from longitude −124° to −116° W in 0.1-degree bins (Figure S2). The ship count per trajectory was calculated by summing the unique ship count in each hourly grid the trajectory intersected, as well as one orthogonally adjacent grid box to either side, in the last 48 h before arriving at Scripps Pier.

Daily regional SST and Chl *a* concentration measurements along trajectories were retrieved (Figure S3 and S4) for trajectories that did not have land overpass in the last 48 h.^74,75^ NOAA SST and Chl *a* are only available daily at 12 PM UTC, and the same daily values were used for all 3-hourly trajectories that occur within a given day.

### 2.5 Source Apportionment

The sources of sulfate aerosol were investigated using two different methods, Multi Linear Regression (MLR) and the tracer method.

#### 2.5.1 Multi Linear Regression (MLR)

The MLR analysis was applied to evaluate the relative contribution from different sources and processes of sulfate aerosols by predicting sulfate mass concentrations (Y) using variables (X_1,_ X_2_, . . .) as predictors (eq 1). Before fitting the MLR model, all variables were standardized using the StandardScaler() function from Python’s scikit-learn package.^76^ The LinearRegression class from scikit-learn was then used to fit the MLR model:
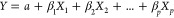
1where *a* is the intercept, and *β*_*i*_(for *i* = 1,···,*p*) are the regression coefficients for the predictor variables *X*_*i*_. The best fit is when the Ordinary Least Squares (OLS) is minimized. After fitting the model, the regression coefficients (*β*_*i*_) were used to derive the relative contributions associated with each variable by comparing the magnitude of each coefficient to the sum of all regression coefficients.^77^

#### 2.5.2 Tracer Method

The tracer method uses the NSS sulfate-to-rBC (NSS SO_4_^2–^/rBC) ratio to identify the sulfate attributable to shipping by scaling the measured rBC by the average ratio when biogenic production of sulfate is lowest, namely during the winter SST minimum.^15,66,78,79^ The shipping-related NSS sulfate is determined as follows:
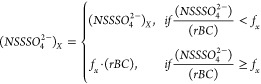
2

Where *f*_*x*_ is the (NSS SO_4_^2–^/rBC) ratio at the SST monthly minimum. The remainder of the NSS sulfate is considered to be biogenic and identified as SST Sulfate.

The fraction of NSS sulfate attributed to shipping emissions is subject to uncertainties due to the variability in emission factors across different ship engines, ship speed (Text. S1), and fuel types.^13,48,80−85^ The tracer method assumes a constant NSS sulfate-to-rBC ratio throughout the year and assumes that all emitted SO_2_ is converted to NSS sulfate during the 24–48 h transport to Scripps Pier. Given these assumptions, the tracer method likely provides an upper bound estimate on the NSS sulfate attributable to shipping emissions.

### 2.6 Cloud Processing Indicators

Two approaches have been used to characterize cloud processing contributions, with one using satellite retrievals of upwind cloudiness and the other using local measurements. Satellite retrievals of cloud properties are produced by NASA Langley Research Center and are based on a family of algorithms referred to as the Satellite ClOud and Radiation Property retrieval System (SatCORPS).^86,87^ For EPCAPE, data from the 18th Geostationary Operational Environmental Satellite (GOES-18) were processed every 30 min using the SatCORPS algorithms, with a pixel resolution of 2 km at nadir. GOES-18 retrievals were further averaged to a 0.5°x0.5° regular grid, with cloud properties averaged according to their cloud top height into low (0–2km), middle (2–6 km), and high (>6 km) cloud properties. Local cloud liquid water path (LWP, g/m^2^) was retrieved by ground-based three-channel microwave radiometers (MWRs) using an advanced retrieval algorithm.^88^ Cloud top height (CTH) and cloud base height (CBH) at Scripps Pier were obtained from the ARM active remote sensing of clouds (ARSCL) value-added data product (VAP).^89−91^ In EPCAPE’s ARSCL data product, the CTH measured at Scripps Pier is described as the top height of hydrometeor layers based on combined Ka-band ARM Zenith Radar (KAZR) and micropulse lidar observations while the CBH is the cloud base best estimate, a combination of ceilometer and micropulse lidar data processing.

#### 2.6.1 Upwind Cloud Vertical Fraction (UCVF)

Since the cloudiness at Scripps Pier and upwind was frequently characterized by complete coverage, UCVF is defined as the extent to which the well-mixed boundary layer is cloud-filled as an observable metric for the period that an air parcel is in cloud. To characterize the most recent influence on the aerosol composition, the average vertical fraction of the boundary layer with upwind clouds was computed for the preceding 24 h of the retrieved back-trajectories. The cloud vertical fraction is the fraction of the Planetary Boundary Layer (PBL) that is occupied by clouds.^92−94^ However, for satellite-retrieved upwind properties, satellite-retrieved CTH was found to be a reasonable approximation of PBL height at Scripps Pier (Figure S3). Therefore, the cloud vertical fraction at each hour along the trajectory was calculated by eq 3 below:
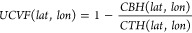
3for each latitude and longitude of the 24 h back trajectory.

Here, the CTH and CBH were taken from the SatCORPS cloud top and base height of the low cloud level category (0–2 km). The SatCORPS CTH at Scripps Pier showed moderate correlation with the CTH reported in ARSCL (r = 0.58, slope = 0.87) (Figure S5a). The SatCORPS CBH showed a moderate correlation (r = 0.48) with the CBH estimated from a ceilometer and KAZR measurements (Figure S5b). The SatCORPS CBH were generally lower compared to the measured CBH by a ratio of 0.47. This discrepancy is likely due to constraints of the VISST algorithm, which may underestimate the CBH of low cloud level since it is derived indirectly from cloud top height and cloud thickness, with the latter parametrized as a function of optical depth.^86,87^

The SatCORPS CTH was used as the Planetary Boundary Layer Height (PBLH) at each hour along the trajectory. SatCORPS CTH at Scripps Pier showed a moderate correlation (r = 0.51) with local PBLH using the Heffter method from radiosonde retrievals,^95,96^ with a slope of 1.0 (Figure S5c). The PBLH from the bulk Richardson number method at Scripps Pier was lower than the PBLH using the Heffter method, showing a slope of 0.43 (r = 0.63) (Figure S 5d), consistent with prior assessments in similar regions.^97,98^ The SatCORPS CTH from low cloud levels at Scripps Pier showed a higher correlation with PBLH using the Heffter method than with the bulk Richardson number method (Figure S5a and S 5d).

A 20% cloud coverage threshold was applied to reduce uncertainty in cloud retrievals by excluding unrealistically low cloud top and base heights due to surface infrared contamination.^86,87^ Cloud vertical fraction at each point was calculated only if the following two conditions were met: 1) the corresponding grid in which the point is located has a cloud coverage higher than 20% and 2) both the trajectory altitude and the retrieved CBH are below the CTH height. If either of these conditions was not met, the value was set to zero. The average UCVF was calculated by averaging the cloud height fraction from the last 24 h of the trajectory.

#### 2.6.2 Liquid Water Content (LWC)

The cloud-mean Liquid Water Content (LWC_mean_, hereafter “LWC”) within the cloud layer at Scripps Pier is calculated using the MWR-retrieved LWP^88^ as well as CTH and CBH from the ARM VAP products.^90^ The LWC (g/m^3^) at Scripps Pier is determined by retrieving the LWP (g/m^2^) and normalizing by the cloud thickness, CTH–CBH (m), as shown in eq 4 below:
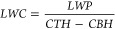
4

A minimum LWP limit of 20 g/m^2^ is applied due to the uncertainty at low cloud LWP.^88^ Only cases with CTH < 2500 m are considered for low-level clouds. To ensure sufficient cloud thickness for accurate LWC calculation, a CTH–CBH difference of greater than 50 m was required.

## 3 Results and Discussion

The objective of this work is to understand the anthropogenic shipping and biogenic phytoplankton sources of SO_2_, as well as the photochemical and aqueous reactions that form aerosol sulfate. Section 3.1 describes the variation of the aerosol composition with the different air masses sampled at Scripps Pier for the 12 months of EPCAPE. The contributions of different regional sources of SO_2_ are apportioned by two methods using rBC as an indicator of shipping emissions and SST as an indicator of biogenic emissions in Section 3.2, the former based on individual tracers and the latter using multiple linear regression (MLR). It is important to note that the MLR method relies on linear relationships to observed variables with their explanatory power limited to the square of the correlation coefficient, which means that the resulting apportionment applies only to the regionally emitted sulfate, as upwind sources are too varied and diluted for their variability to be explained by contemporaneous observations. Since aerosol sulfate is formed as a secondary product in the atmosphere, the processes by which SO_2_ then forms aerosol sulfate are investigated using metrics for photochemical and aqueous processes. These sulfate formation processes are considered independently of the sources in Section 3.3 using indicators of photochemical radiation (DSW) and aerosol and cloud aqueous reactions (RH and UCVF), which is justified based on the weak correlations of the source variables (rBC and SST) to the process variables (DSW, RH, and UCVF), as shown in Table S5.

### 3.1 Air Mass Characterization and Aerosol Composition

Back trajectories were used to identify air masses from consistent source regions in order to attribute NSS sulfate mass concentration at Scripps Pier to marine phytoplankton and shipping sources. Linear regression correlations were used to interpret the relationships between variables, classifying the strength of correlations as follows: 0.1–0.29 as weak, 0.3–0.69 as moderate, and 0.7–0.9 as strong.^99^ A correlation was considered significant for p-value below 0.05.^100,101^

**Figure 1:**
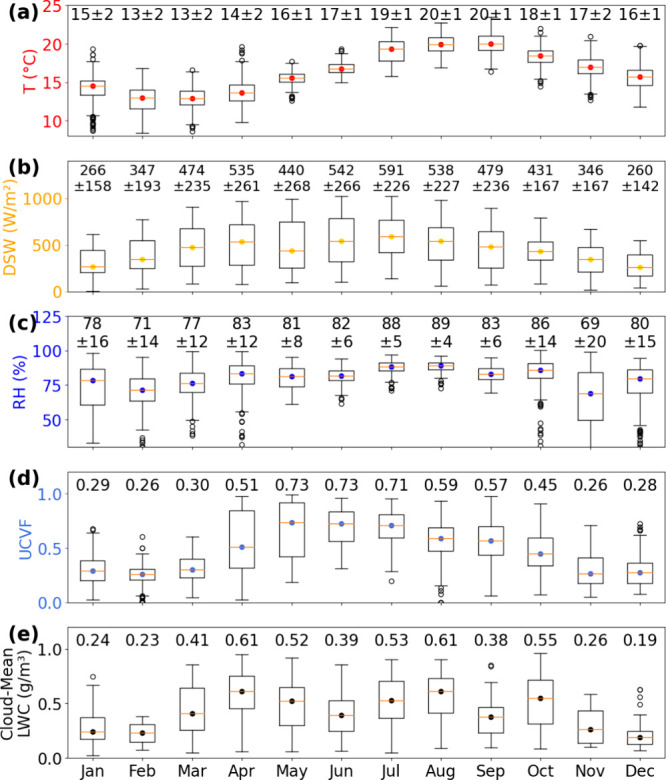
Monthly boxplots of meteorological conditions during EPCAPE: (a) the average ambient air temperature (°C), (b) the downwelling shortwave radiation (DSW), (c) the average relative humidity (RH) (%), (d) the average upwind cloud vertical fraction (UCVF), and (e) the local cloud-mean liquid water content (LWC) (g/m^3^). (a) and (c) were retrieved and calculated from AOSMET at Scripps Pier, La Jolla, California. (e) was computed by utilizing the Minnis cloud products using VISST algorithm based on the trajectory starting at Scripps Pier and (b) and (d) were retrieved using the local measurements at Scripps Pier combining microwave radiometers and ARSCL retrievals.

Ambient air temperature was highest in summer months from July to September (20 ± 2 °C), with a 12-month average and standard deviation of 16 ± 3 °C (Figure 1a). The observed DSW peaked in July with monthly average and standard deviation of 591 ± 226 W/m^2^, compared to the 12-month average and standard deviation of 448 ± 180 W/m^2^ (Figure 1b). The 12-month average RH was 79 ± 14%, and the monthly average RH remained consistently above 80% from April to October with low standard deviation (84 ± 9%) (Figure 1c). From November to March, the observed RH was lower and showed more variation (72 ± 17%) (Figure 1c). Precipitation events occurred throughout the year but were most frequent in February and March (Figure S6b). Precipitation periods were removed from the analysis to minimize the effects of removal by wet scavenging.^102,103^

**Figure 2:**
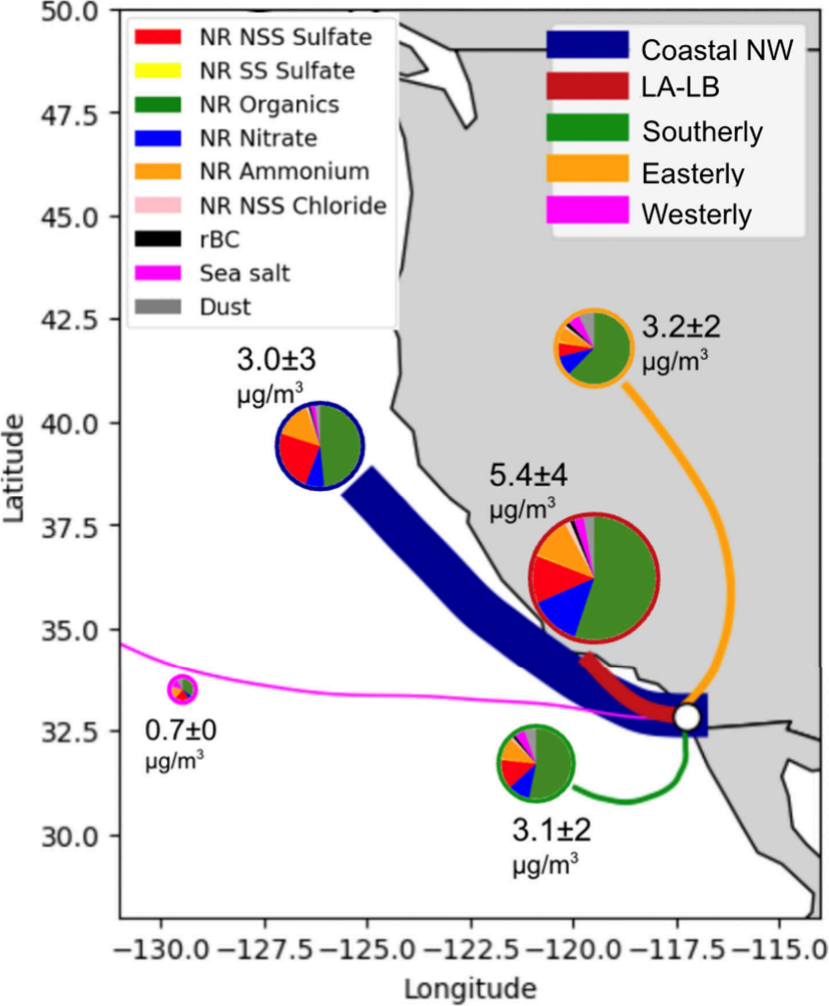
Five identified transport clusters during EPCAPE with their PM1 mass fractions: 1) Coastal NW cluster (64%, Blue), 2) LA-LB cluster (18%, Red), 3) Southerly (6%, Green), 4) Easterly (9%, Orange), and 5) Marine Westerly (3%, Magenta). The cluster legend is shown on the top right and the cluster trajectory thickness represents the occurrence fraction of each cluster relative to the observed trajectories. The pie charts show each cluster’s PM1 aerosol composition as mass fractions with the average total PM1 mass concentrations (μg/m^3^) labeled in adjacent text. The PM1 composition legend (NR NSS sulfate (red), NR SS sulfate (yellow), NR organics (green), NR nitrate (blue), NR ammonium (orange), NR NSS chloride (light pink), rBC (black), sea salt (magenta) and dust (gray)) is shown on the top left.

**Figure 3:**
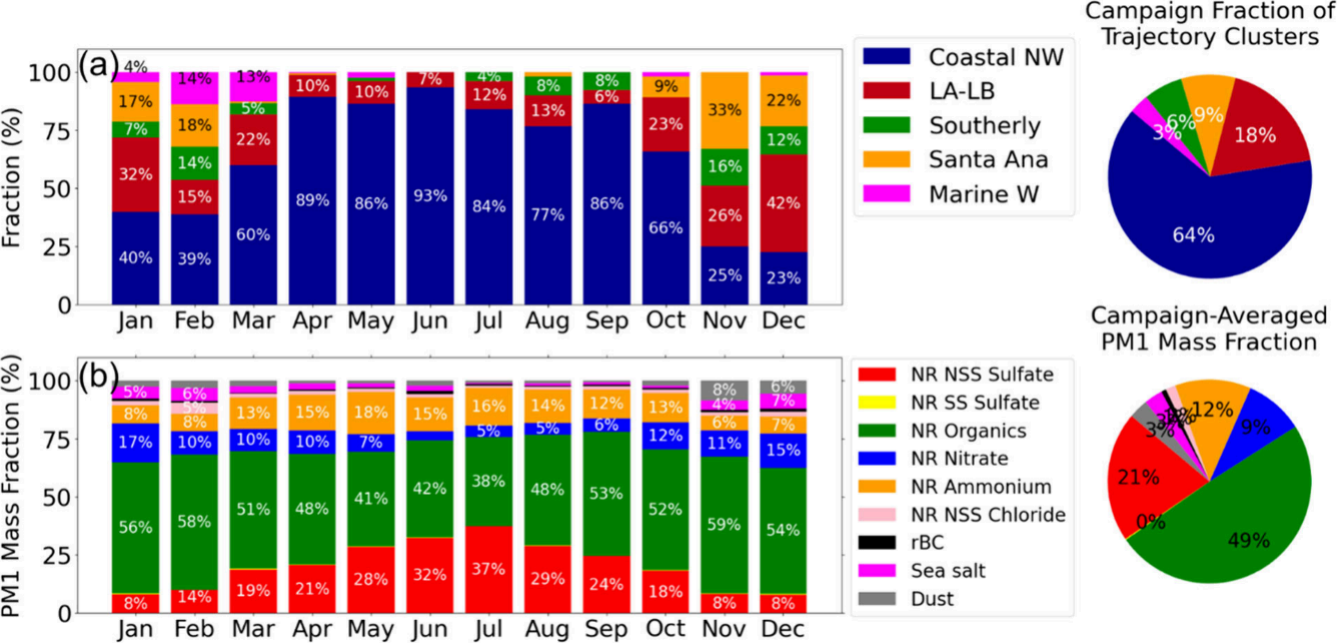
(a) Monthly average fraction of transport cluster occurrence, accompanied by a pie chart illustrating the campaign occurrence fraction of each cluster relative to the observed trajectories. (b) PM1 aerosol mass concentrations, including NR NSS sulfate (red), NR SS sulfate (yellow), NR organics (green), NR nitrate (blue), NR ammonium (orange), NR NSS chloride (light pink), rBC (black), sea salt (magenta) and dust (gray), with a pie chart on the bottom right showing the annual averaged PM1 mass fraction.

The air mass trajectories during EPCAPE were separated into five clusters using a k-means clustering algorithm.^50,73^ The five clusters are characterized as (1) coastal northwesterly air masses following the coast of California, representing 64% of trajectories (CNW), (2) slower air masses coming from Los Angeles and Long Beach regions, representing 18% of trajectories (LALB), (3) southerly air masses, representing 6% of trajectories (SOU), (4) continental northeast/easterly air masses from San Bernardino and Riverside areas, representing 9% of trajectories (EAS), and (5) westerly air masses from over the ocean, representing 3% of trajectories (MWE) (Figure 2 and 3a).

CNW trajectories occurred more than 75% of the time from April to September (Figure 3a). From November to February, more trajectories passed over urban and continental regions as part of the LALB, EAS, and SOU clusters. These urban-influenced trajectories occurred frequently during November and December, accounting for 75–77% of trajectories at Scripps Pier with only 23–25% as CNW (Figure 3a).

The largest component of the submicron aerosol for each transport cluster was NR organic, which contributed an annual average of 49% of PM1 mass with average concentration of 1.9 ± 1.8 μg/m^3^ (Figure 3b). The other components of the annual average PM1 mass were NR sulfate at 21% (0.78 ± 0.7 μg/m^3^), NR ammonium at 12% (0.46 ± 0.5 μg/m^3^), NR nitrate at 9% (0.37 ± 0.6 μg/m^3^), dust at 3% (0.11 ± 0.1 μg/m^3^), sea salt at 3% (0.11 ± 0.1 μg/m^3^), rBC at 1% (0.04 ± 0.0 μg/m^3^) and NR NSS chloride at 1% (0.03 ± 0.0 μg/m^3^) of annual average (Figure 3b). SS sulfate accounted for only 2% of the total NR sulfate concentrations, with the remaining 98% classified as NSS sulfate.

The highest monthly average NSS sulfate mass concentrations for EPCAPE were in July (2.00 ± 0.8 μg/m^3^) and August (1.25 ± 0.5 μg/m^3^) (Table. S1, Figure 3b). These NSS sulfate mass concentrations are substantially lower than previous studies at Scripps Pier during July-August 2008 (3.0 ± 0.8 μg/m^3^)^51^ and August-September 2009 (3.1 ± 1.7 μg/m^3^).^50^ NSS Sulfate mass concentration was lowest during January-February, with average concentrations of 0.28 ± 0.3 μg/m^3^ and 0.25 ± 0.2 μg/m^3^ (Table. S1). These mass concentrations are lower than a previous study at the same site in February-March 2009 (1.5 ± 0.8 μg/m^3^).^49^ While the decrease in summer concentrations of sulfate may be associated with interannual variability in biogenic emissions, the wintertime decreases of more than 80% is likely associated with the 2015 and 2020 implementations of reduced fuel sulfur regulations in California-regulated waters.^32,33,104,105^

Both sea salt and dust mass concentrations were higher during November-December than January-October. Sea salt concentrations were 0.21 ± 0.3 μg/m^3^ in November and 0.35 ± 0.3 μg/m^3^ in December. Dust concentrations also increased during this period, with concentrations of 0.43 ± 0.8 μg/m^3^ in November and 0.30 ± 0.2 μg/m^3^ in December (Table. S1, Figure 3b). The coincident increase in both sea salt and dust concentrations during November and December may be associated with EAS back-trajectories, typified by air masses traveling near the Salton Sea basin, a region previously identified as a source of both dust and sea salt (Figure 2 and 3).^106−108^

The NSS sulfate mass concentration and mass fraction were highest for the CNW cluster (0.90 ± 0.7 μg/m^3^ per trajectory, 28%) with lower concentrations for the LALB (0.77 ± 0.6 μg/m^3^ per trajectory, 13%), SOU (0.45 ± 0.5 μg/m^3^ per trajectory, 15%) and EAS (0.23 ± 0.2 μg/m^3^ per trajectory, 7%) trajectories (Figure 2). The high NSS sulfate contribution in the CNW cluster back-trajectories suggested more NSS sulfate coming from marine-related sources. The frequent occurrence of CNW back-trajectories translates to these air masses accounting for 76% of the annual NSS sulfate.

### 3.2 Apportionment of Submicron Sulfate by SO_2_ Source

**Figure 4:**
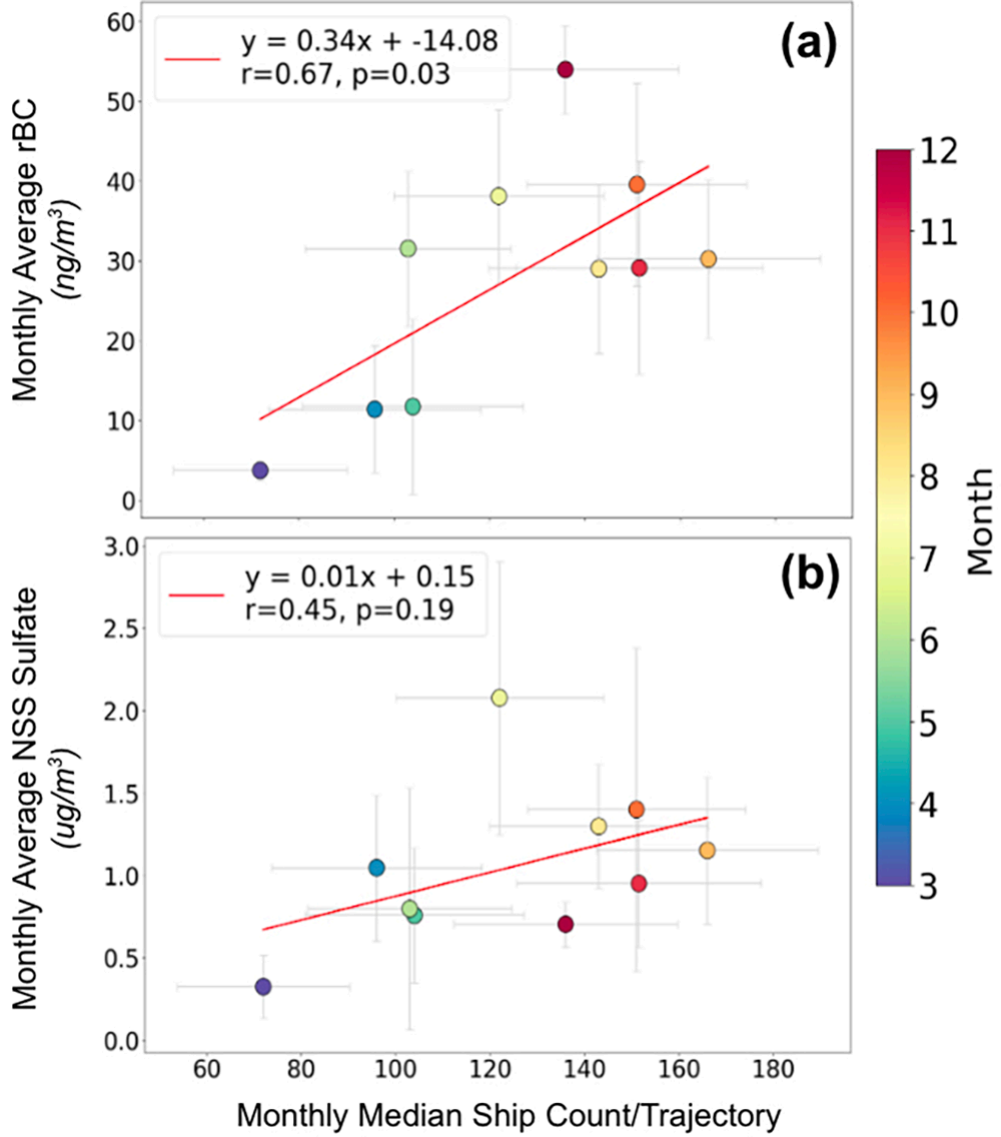
Scatter plots and least-squares linear fits showing the relationship between the monthly median ship count per trajectory with (a) the monthly average rBC mass concentration (ng/m^3^) and (b) the monthly average NSS sulfate mass concentration (μg/m^3^). Data points are colored by month, and error bars are included. January and February 2024 were not shown as they are not available from AIS traffic data.

The two sources of SO_2_ that contribute to NSS sulfate formation in coastal Southern California are marine biogenic emissions from phytoplankton and anthropogenic emissions from sulfur-containing fuel used by ships. Ship emissions offshore of coastal Southern California mainly consist of rBC, NSS sulfate, and nitrate.^48,82−84,109,110^ The moderate correlation between monthly median ship count per trajectory and monthly average rBC concentrations (r = 0.67, *p* < 0.05, Figure 4a) suggests that the monthly trends in rBC observed at Scripps Pier are likely associated with ocean-going vessels in the region. The poor positive correlation (r = 0.07, *p* < 0.05) in the 3-h resolution data indicates that air mass mixing and ship count uncertainties obscure the relationship at finer temporal scales (Table S2).

The stronger dependence of rBC on regional shipping activities compared to NSS sulfate and nitrate is observed at both monthly and 3-h resolution, likely because rBC is a primary and relatively conservative tracer for emissions from ships whereas NSS sulfate and nitrate form by secondary reactions that limit their correlations with ship count.^82−84,109^ Given the ship-to-ship variability in emissions, it is not surprising that the correlations between the monthly median ship count per trajectory and the monthly average NSS sulfate (Figure 4b) and nitrate mass concentrations were not statistically significant (*p* > 0.05). The lack of correlation between ship count and nitrate mass concentrations is likely due to the lower amounts of nitrate emitted from ship emissions compared to NSS sulfate, which are approximately ten times lower,^82,110^ as well as confounding contributions from urban nitrate sources.

**Figure 5:**
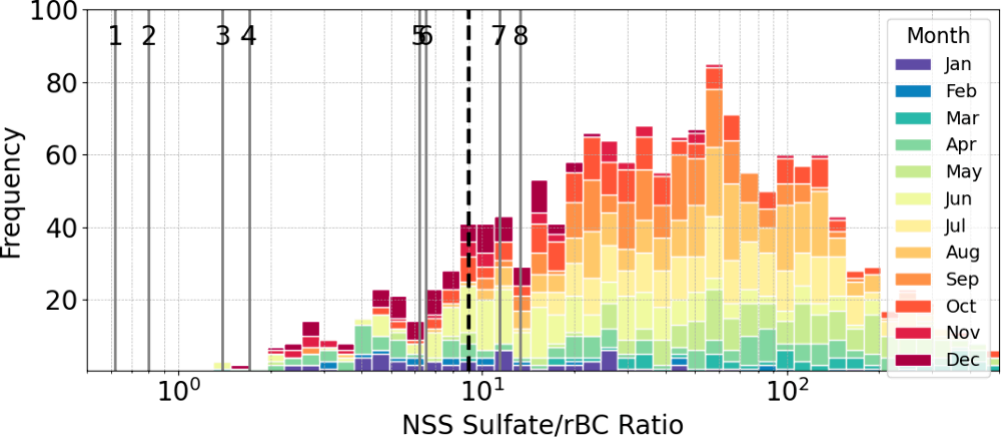
Frequency distribution of NSS sulfate/rBC ratios from the Coastal NW transport cluster, overlaid with NSS sulfate/rBC ratios reported from ship plumes in previous measurements off the California coast. The median and standard deviation of NSS sulfate/rBC ratios observed at Scripps Pier were 40 ± 80 during the Coastal NW cluster. The labels indicate ship plume characteristics, with corresponding number labels: (1) 0.62: Marine Diesel Oil (MDO) (0.07% sulfur content, speed 12 knots); (2) 0.8: MDO (0.1% sulfur content, speed 12 knots); (3) 1.4: Ultralow-sulfur diesel (ULSD) at 1600 rpm; (4) 1.7: MDO (0.1% sulfur content, speed 6.9 knots); (5) 6.2: MDO (0.1% sulfur content, speed 2.9 knots); (6) 6.4: Hydrogenation-Derived Renewable Diesel (HDRD) at 1600 rpm; (7) 11.3: HDRD at 700 rpm; (8) 13.2: Polluted marine atmosphere. References: (1) Lack et al., 2011; (2), (4), (5) Cappa et al., 2014; (3), (6), (7), (8) Price et al., 2017. The dashed black line at a ratio of 9.0 represents the baseline NSS sulfate/rBC ratio used in this study to estimate NSS sulfate from shipping emissions.

The median and standard deviation of NSS sulfate/rBC ratios observed at Scripps Pier were 40 ± 80 during the Coastal NW cluster (Figure 5). The lowest monthly median NSS sulfate/rBC ratio of 9.0 was observed in December. During the warmer months from April to August, NSS sulfate/rBC ratios were relatively high with monthly medians above 50, except for June which had monthly median of 18. Previous studies have reported a range of approximately 0.6 to 11.3 for median NSS sulfate/rBC ratios measured from fresh ship plumes with different fuel types, vessel speeds, and engine speeds in this region, as noted in Figure 5.^81−84^ Price et al.^84^ reported a median NSS sulfate/rBC ratio of 13.2 for polluted marine conditions off the coast of Southern California, which is higher than the winter months during EPCAPE. The observed higher range of NSS sulfate/rBC ratios at Scripps Pier (Figure 5) compared to the reported ratios from ship plumes and polluted marine conditions suggested that rBC from shipping had also decreased because of cobenefits from fuel sulfur reductions.^81,82,84,111^

Variations in biogenic sulfate emissions off the Southern California coast have been associated with seasonal changes in sea surface temperature (SST), wind speed, and Chl *a*.^21,112,113^ The correlation between NSS sulfate mass concentrations and local SST at Scripps Pier was moderate (r = 0.50), with the correlation between NSS sulfate concentrations and retrieved SST along the trajectory being slightly weaker (r = 0.40) (Table S2). The lower correlation to retrieved SST is likely associated with uncertainties and limited time resolution of the retrievals along the trajectories based on the limited agreement between the local and retrieved SST measurements at Scripps Pier (r = 0.7, slope = 0.62, Figure S7b). Wind speed did not show a significant correlation with NSS sulfate mass concentrations (Table S2). Chl *a* concentration provides another indicator of biogenic ocean productivity,^112,113^ but neither local measurement at Scripps Pier nor retrieved Chl *a* concentration along the trajectory had a significant correlation with NSS sulfate mass concentrations (Table S2).

#### 3.2.1 MLR of NSS Sulfate by SO_2_ Sources

**Figure 6:**
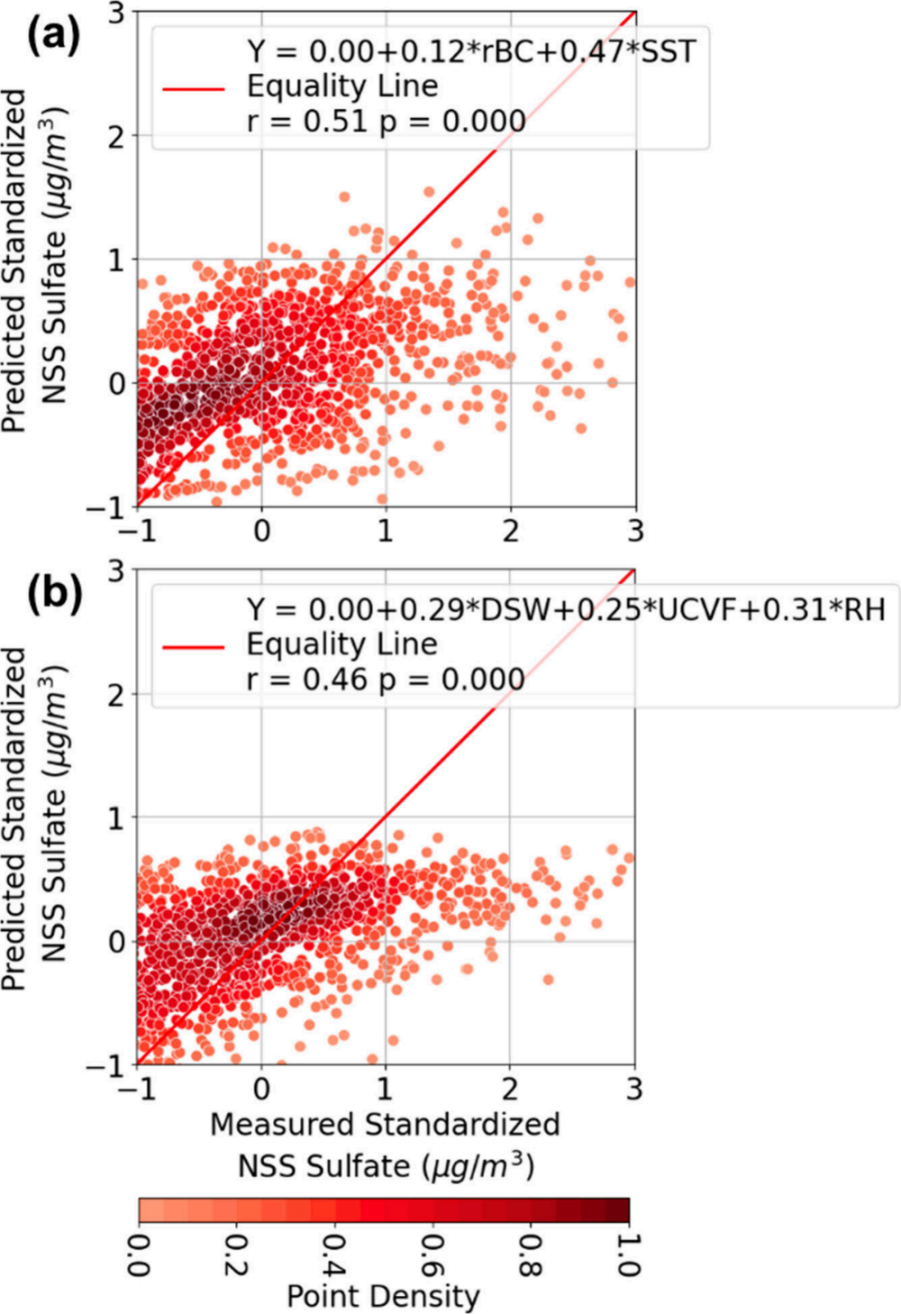
Scatter plots comparing the measured standardized regionally emitted NSS sulfate mass concentrations (μg/m^3^) against the predicted standardized NSS sulfate mass concentrations (μg/m^3^) from MLR models: (a) NSS sulfate (rBC, SST) and (b) NSS sulfate (DSW, UCVF, RH) using the data points from the CNW transport cluster.

**Figure 7:**
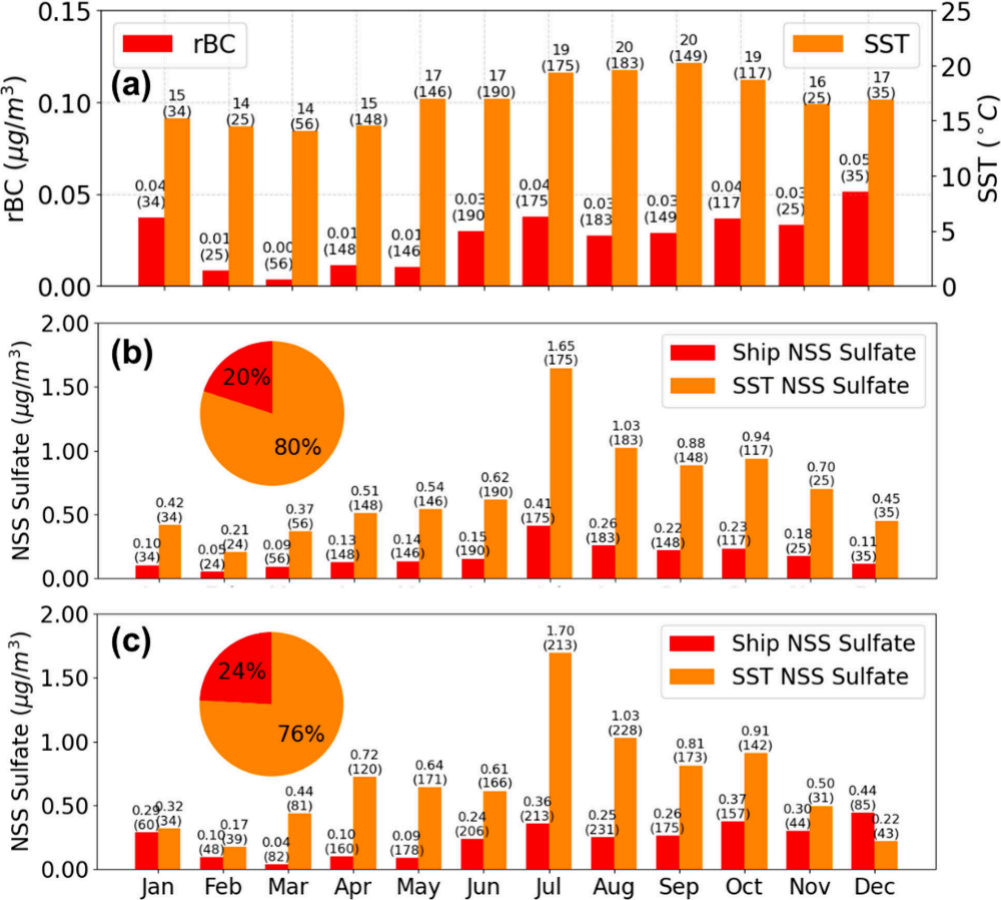
(a) Monthly average rBC mass concentrations (μg/m^3^) (red, left *y*-axis) and SST (°C) (orange, right *y*-axis) per trajectory, with different scales on the *y*-axes. (b) Comparison of monthly average regionally emitted NSS sulfate mass concentrations (μg/m^3^) for source apportionment between NSS sulfate from ships (red) and SST (orange) using MLR analysis based on the CNW transport cluster. (c) Comparison of monthly average NSS sulfate from ships (red) and SST (orange) using the rBC-tracer method. Pie charts in (b) and (c) display the annual mass fractions of ship and SST NSS sulfate. Mean values are shown above each bar, with sample size in parentheses.

Multiple linear regression (MLR) was used to distinguish the contributions of the many factors that contribute to NSS sulfate sources. The two indicators of SO_2_ sources that had the strongest correlation (r = 0.51, *p* < 0.05) were (1) rBC mass concentration for shipping emissions and (2) SST for marine biogenic emissions (Figure 6a, Table S3), together explaining 26% of the sulfate variability. The coefficients of the MLR were used to calculate the mass fraction of regionally emitted ship NSS sulfate from the rBC and regionally emitted biogenic NSS sulfate from the SST. Over the 12-month period, an approximate annual NSS sulfate contribution for regional sources of only 20% is associated with shipping emissions and 80% associated with SST. Monthly ship NSS sulfate per trajectory remained lower than 0.50 μg/m^3^ in all months. Monthly SST NSS sulfate varied substantially as it constituted most of the mass fraction of NSS sulfate, from as low as 0.21 μg/m^3^ in February to peaks of 1.65 μg/m^3^ in July and 1.03 μg/m^3^ in August. The weak but significant correlation between rBC and SST (r = 0.17, *p* < 0.05) also shows covariability between rBC and SST (Table S3), making rBC and SST nonindependent contributors to NSS sulfate concentrations (Figure 7a). The monthly average SST per trajectory peaked during the summer from July to September, reaching 19–20 °C, coincident with the NSS sulfate peak (Figure 7a). During fall and winter months, both SST and NSS sulfate decreased (Figure 7a). Consequently, the MLR method supports the attribution of 78–82% of the regionally emitted NSS sulfate concentrations to SST-associated biogenic emissions.

This MLR method is limited to regionally emitted sulfate sources and does not account for upwind sources that have been diluted or mixed over multiple days of transport. For example, volcanic eruptions at Kı̅lauea, Hawaii, were reported on 5 January -7 March, 7–19 June, and 10–15 September in 2023.^114−117^ However, 10-day back trajectories showed that air masses did not pass near Hawaii during these periods (Figure S9) and residuals relative to our rBC-based model were not enhanced in NSS sulfate (Figure S10a), indicating the Kı̅lauea eruptions had minimal impact on the variability of the regionally emitted SO_2_.

#### 3.2.2 NSS Sulfate Apportionment to SO_2_ Sources by the rBC Tracer Method

rBC provides an appropriate tracer for shipping-related emissions because it is emitted during fuel combustion as a primary aerosol constituent and is nonreactive.^48,82,84^ While the ship fleet and the associated ratios of sulfate to rBC vary, the use of rBC as a tracer for shipping sulfate is supported by the moderate correlation (r = 0.67, *p* < 0.05) between the monthly average rBC and the monthly median ship count for CNW trajectories over the ocean (Figure 4a). Coastal vehicles, trucks, and fires can also contribute to rBC variability,^51,109,110^ reducing the correlation between monthly average rBC with monthly median shipping per trajectory (Figure 4a). The tracer method provides an independent estimate of SO_2_ source contributions to NSS sulfate, although it likely should be interpreted as an upper bound on shipping emissions because of the assumption that the wintertime ratio of sulfate to rBC is completely attributable to shipping.^15,66,78^ It also assumes that a constant sulfate to rBC ratio is representative of shipping emissions throughout the year. The minimum monthly median of NSS sulfate/rBC ratios provides the baseline for background shipping emissions, which was 9.0 in December (Figure 5, dashed line).

Applying this ratio for the tracer method (section 2.5.2) results in a similar estimated annual NSS sulfate contribution from shipping emissions of approximately 24% (compared to 20% by the MLR method), with the remaining 76–80% attributed to biogenic emissions associated with SST (Figure 7c). Monthly ship NSS sulfate from the tracer method was larger and more variable (0.22 ± 0.1 μg/m^3^) than the MLR method (0.17 ± 0.1 μg/m^3^), ranging from 0.04 μg/m^3^ in March to 0.44 μg/m^3^ in December, because of the month-to-month variability in rBC. SST NSS sulfate represented the difference between the measured NSS sulfate and the rBC-associated sulfate, which showed seasonal patterns similar to those observed with the MLR method, with the highest contributions in July and August. The tracer method peak concentrations in SST NSS sulfate were comparable to the MLR method at 1.70 μg/m^3^ in July and 1.03 μg/m^3^ in August.

Overall, the two methods for separating regionally emitted ship and biogenic NSS sulfate both identified marine biogenic sulfur as the majority contribution, with MLR providing a lower bound for shipping due to its moderate correlation with rBC concentrations and the tracer method providing an upper bound because of its neglect of wintertime biogenic sulfate. The resulting range of 20% to 24% contribution of SO_2_ from shipping emissions to NSS sulfate mass concentration is within the 10–44% range of NSS sulfate measurements at Scripps Pier in 2008 (for particles below 1.5 μm).^52^ The lower shipping contribution in 2023–24 compared to 2008 is expected given the mandated 2015 reduction in ship sulfur content fuel from 1.0% to 0.1% in the sulfur emissions control area, which includes the IMO designated waters within 370 km of the United States.^32,33,104,105^

### 3.3 Photochemical, Cloud, and Aerosol Aqueous Sulfate Formation

Sulfur dioxide (SO_2_) can be oxidized to form sulfate by two types of pathways – photochemical and aqueous reactions. Photochemical reactions can oxidize SO_2_ to sulfate in the gas phase during cloud-free conditions or when liquid water availability is limited.^1,37^ Aqueous reactions are known to increase the rate and yield of forming aerosol sulfate from gas-phase precursors.^1,5,37,38,118,119^ The relative contributions of these reactions to forming sulfate from SO_2_ depend on the availability of direct sunlight and both aerosol and cloud liquid water properties during airmass transit to Scripps Pier where sulfate is measured. Prior work has relied primarily on local measurements of RH and cloud LWC as indicators of upwind conditions.^35,40,44,120^ However, back-trajectories based on reanalyses also provide estimates of upwind conditions.^71,72^ The influence of the UCVF provides an estimate of upwind cloud processing time and of the measured cloud LWC as an indicator of local in-cloudwater amount to the influence of local RH as an indicator of aerosol water. NSS sulfate showed moderate correlations to process indicators in several months during EPCAPE, providing more consistency in the explained variability than for organic or nitrate components (Table S6). The analyses are limited to CNW back-trajectories that did not pass over land or include precipitation within the previous 24 h to remove variability associated with other sources and sinks.

The monthly median UCVF was above 0.5 from April to September (Figure 1d) when the air mass trajectories typically traveled at altitudes below the upwind CTH (700 ± 300 m) and above the upwind CBH (150 ± 200 m) (Figure S6c). The high UCVF (>0.6) with trajectories below CTH and low upwind CBH suggest that upwind trajectories remained consistently within the cloud geometrical boundaries, providing frequent cloud processing. The UCVF decreased from July to September and remained low (monthly median UCVF < 0.6) from September to March (Figure 1d). From September to March, upwind CBH was 370 ± 300 m and trajectory altitudes exceeded 500 m, sometimes traveling above upwind CTH (Figure S6c). Both the lower UCVF and above-cloud trajectories are consistent with less cloud processing from September to March.

The local cloud-mean LWC increased from April to July, with monthly median LWC consistently above 0.2 g/m^3^, and a median cloud-mean LWC of 0.29 g/m^3^, peaking in May consistent with the high LWP observed during May (130 ± 120 g/m^2^) (Figure S6d). In December and January, despite low UCVF values (<0.6), higher LWC levels with monthly medians >0.3 g/m^3^ were observed. This increase in LWC corresponded to thinner cloud layers, with median thicknesses of 230 m in December and 260 m in January, compared to the annual median of 400 m (Figure S6e). The filtering criteria removed 45% of measurements with local LWP < 20 g/m^2^ and 62% of measurements with local CTH > 2500 m, resulting in fewer than 50 3 h measurements per month for October-March, effectively excluding them from this analysis (Figure S6d). April to September had consistently high local LWP (monthly median >50 g/m^2^) with 80% of local CTH measurements below 2500 m and cloud thickness greater than 50 m (Figure S6e).

**Figure 8:**
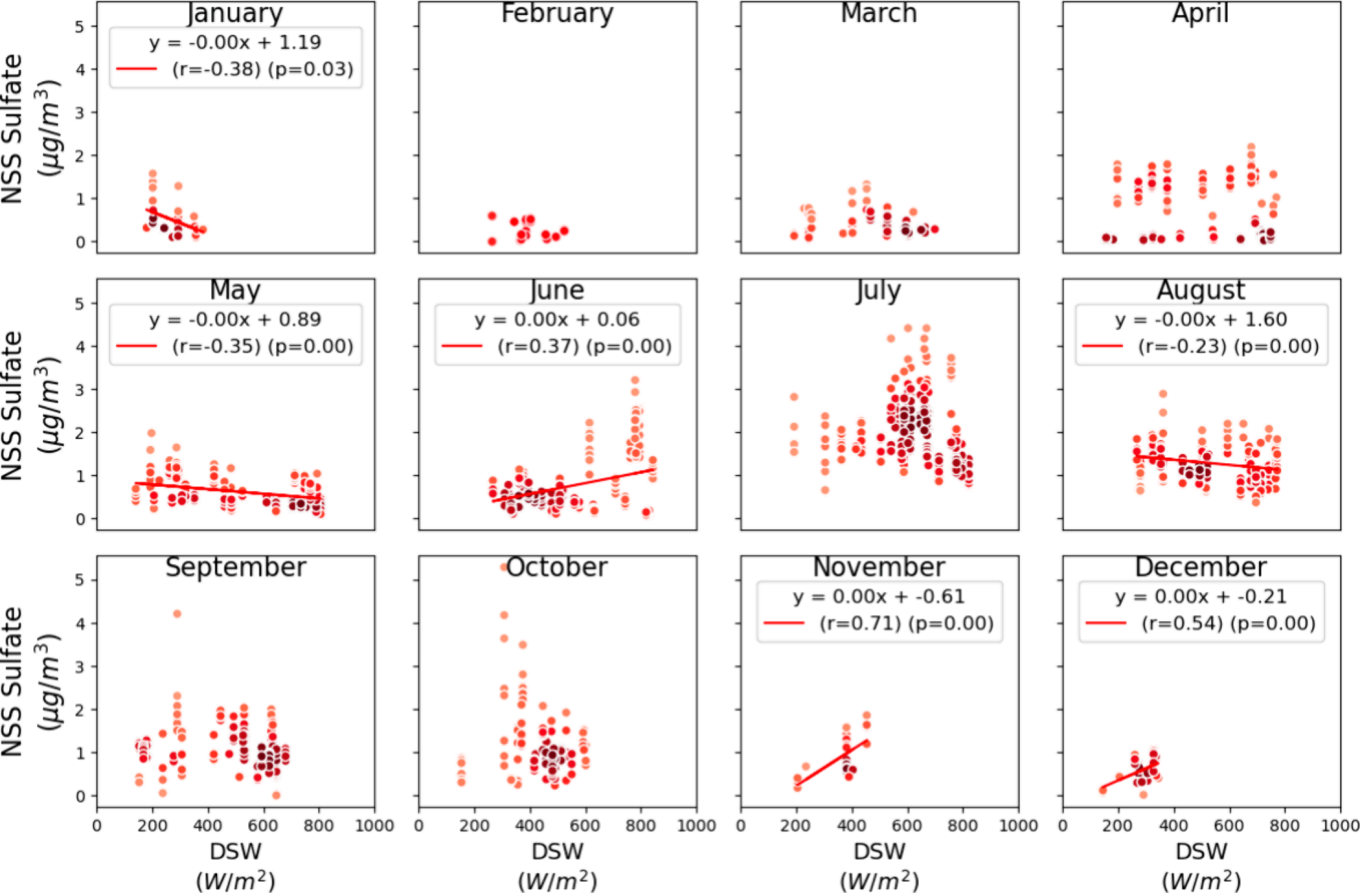
Monthly point-density scatter plots of NSS sulfate mass (μg/m^3^) vs Downwelling Shortwave Radiation. Linear regression lines are fitted where significant correlations are observed (*r* > 0.2 and *p* < 0.05), with the corresponding regression equation, Pearson correlation coefficient (r), and p-value indicated on the plots. The color darkness represents the relative point density.

**Figure 9:**
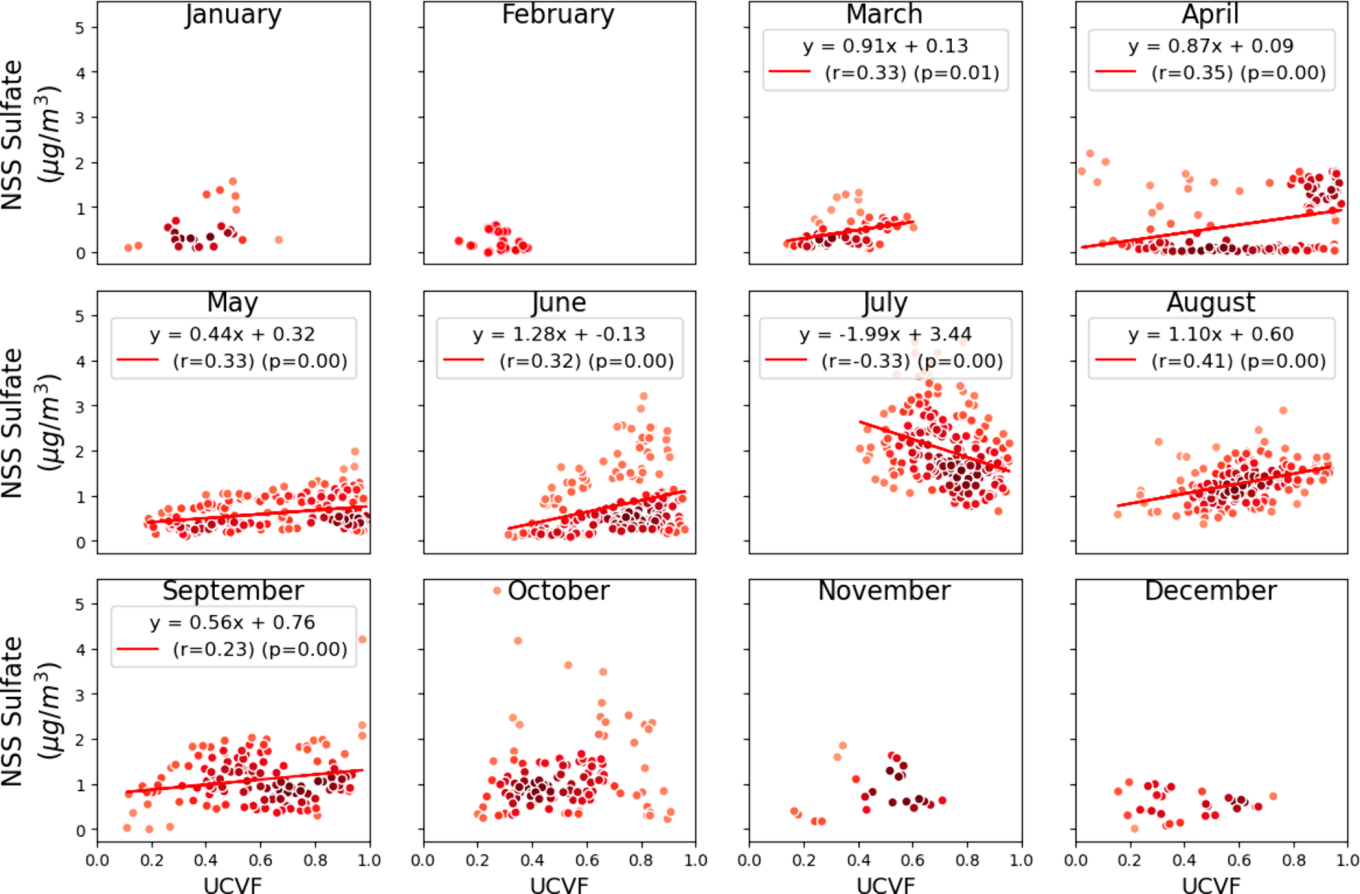
Monthly point-density scatter plots of NSS sulfate mass (μg/m^3^) vs UCVF. Linear regression lines are fitted where significant correlations are observed (*r* > 0.2 and *p* < 0.05), with the corresponding regression equation, Pearson correlation coefficient (r), and p-value indicated on the plots. The color darkness represents the relative point density.

Moderate to strong correlations between NSS sulfate mass concentrations and DSW were significant during low DSW months, including November (r = 0.71) and December (r = 0.54), as well as in June (r = 0.37) as it transitioned into July when DSW was observed to be highest (Figure 8, Figure 1b). Moderate correlations between NSS sulfate mass concentrations and UCVF were significant during months when UCVF was high (>0.5), including April (r = 0.35), May (r = 0.33), June (r = 0.33), and August (r = 0.41) (Figure 9). These correlations provide evidence of upwind cloudiness contributing to secondary NSS sulfate formation during transport over the ocean in cloudy months. The percent increase in NSS sulfate mass concentration ranged from 50% to 260% when UCVF increased from 0.2 to 1 for April, May, August and September, with a significantly higher increase observed in June (Figure 9). The observed range of 50–260% increases in NSS sulfate mass during April, May and August are within the range of previous reports of 25–220% increases in NSS sulfate mass due to cloud processing in observational and modeling studies.^35,40,42,44^ Different parameters have been used to define cloud conditions, including minimum cloud-mean LWC values ranging from 0.01 to 0.02 g/m^3^ to 100 mg/m^3^, RH thresholds of 94–97%, and minimum cloud durations of 40 min to 4 h.^35,40,42,44^

In contrast to prior studies, local cloud LWC showed no significant correlations with NSS sulfate, instead having weak negative correlations in April and August (r = −0.38 and r = −0.32, respectively, with *p* < 0.05) (Figure S11). The lack of positive correlation suggests that sulfate yields were not sensitive to the amount of water available in clouds, which is expected if SO_2_ was already depleted or water was in excess. The negative significant correlations during April and August could indicate in-cloud scavenging of submicron NSS sulfate to supermicron particles, as observed by Gilardoni et al.^120^ These EPCAPE results are different from studies that found increases in NSS sulfate mass concentrations with local LWC, including Ge et al.^40^ who observed a 100% increase when LWC exceeded 0.02 g/m^3^ and Harris et al.^44^ who noted increases of 20%–200% when LWC was above 0.1 g/m^3^. Likely the distinction between EPCAPE and these prior studies is the larger role of upwind cloudiness over local cloudiness in the EPCAPE CNW back-trajectories. Both cloudwater and cloud fraction are important for aqueous reactions, as cloudwater can enhance the rate of water uptake,^40,44,45^ but cloud vertical fraction may better represent the duration and frequency of cloud events, which control sulfate formation if liquid water is in excess.

**Figure 10:**
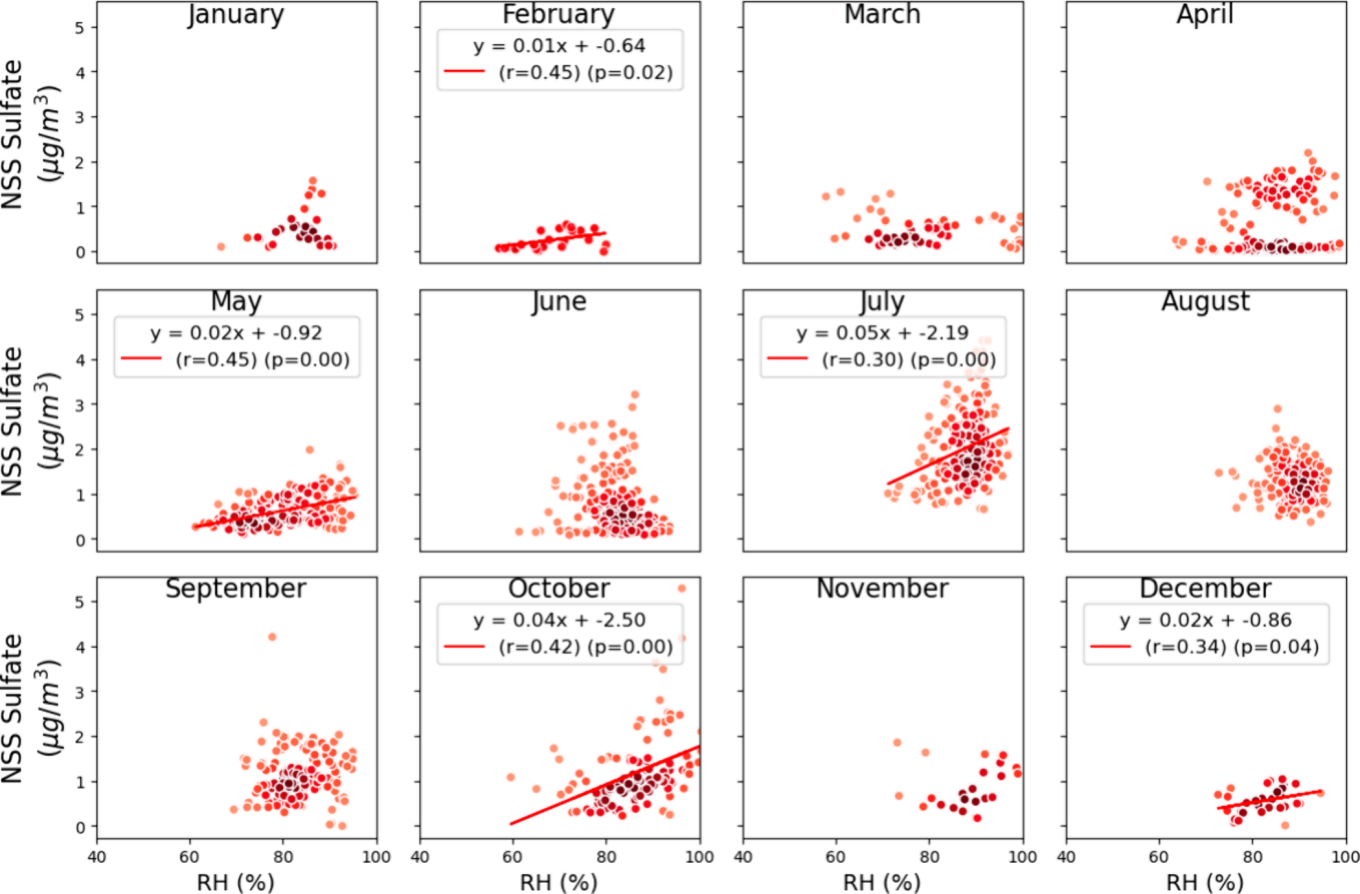
Monthly point-density scatter plots between NSS sulfate mass (μg/m^3^) and the measured RH (%) at the Scripps Pier. Linear regression lines are fitted where significant correlations are observed (*r* > 0.2 and *p* < 0.05), with the corresponding regression equation, Pearson correlation coefficient (r), and p-value indicated on the plots. The color darkness represents the relative point density.

September through January had lower amounts of upwind low clouds with UCVF < 0.5 and showed weak to moderate significant correlations between NSS sulfate mass and RH, with r values ranging from 0.10 to 0.42 (Figure 10). During October to January, the wider range of variation in RH levels with standard deviations exceeding 10% (74 ± 18%) allowed the observed correlations during these months to be significant (Figure 1). May and July also showed a moderate positive significant correlation (r = 0.30–0.45, *p* < 0.05). These results are consistent with observations from other studies,^7,10,121^ which reported RH-induced sulfate formation under weather conditions with RH variation (30–70%).

The high cloudiness during April to August had average daily RH at Scripps Pier that remained consistently above 80% (84 ± 9%) and lacked correlations between NSS sulfate and RH because of the low RH variability (Figure 1). In July, when average UCVF > 0.5 and RH > 80%, there was a significant negative correlation (r = −0.33 and *p* < 0.05) to UCVF (Figure 9) but positive significant moderate correlation with RH (r = 0.30 and *p* < 0.05) (Figure 10). Likely this is because RH and UCVF were negatively correlated to each other in July, so that any positive contribution from UCVF was overwhelmed by the correlation of sulfate to RH. February and March also did not show correlations of NSS sulfate to UCVF or RH due to the high frequency of precipitation in the preceding 24 h (Figure S6b)

The dependence of NSS sulfate mass concentration on RH ranged from 0.01 to 0.05 NSS sulfate mass concentration (μg/m^3^) per 1% increase in RH. Relative to the NSS sulfate mass at 80% RH, this represents a rate ranging from 3% to 6% NSS sulfate mass increase per%RH. The mass concentration dependence on RH is significantly lower than the slope of 0.6 μg/m^3^ per%RH observed in the more polluted region of Beijing, China,^121^ likely due to the much lower concentrations of SO_2_ available at EPCAPE. However, the relative increase in NSS sulfate mass is comparable to the 3% to 9% NSS sulfate mass increase per RH percentage observed for Beijing and Nanjing, China.^7,10,121,122^ In addition, SO_2_ uptake and aqueous oxidation depend on pH,^1^ so the lower increases in sulfate concentration with RH for EPCAPE may reflect the lower range of ammonium to sulfate relative to urban China in addition to the lower SO_2_ emissions (Table S7).

**Figure 11:**
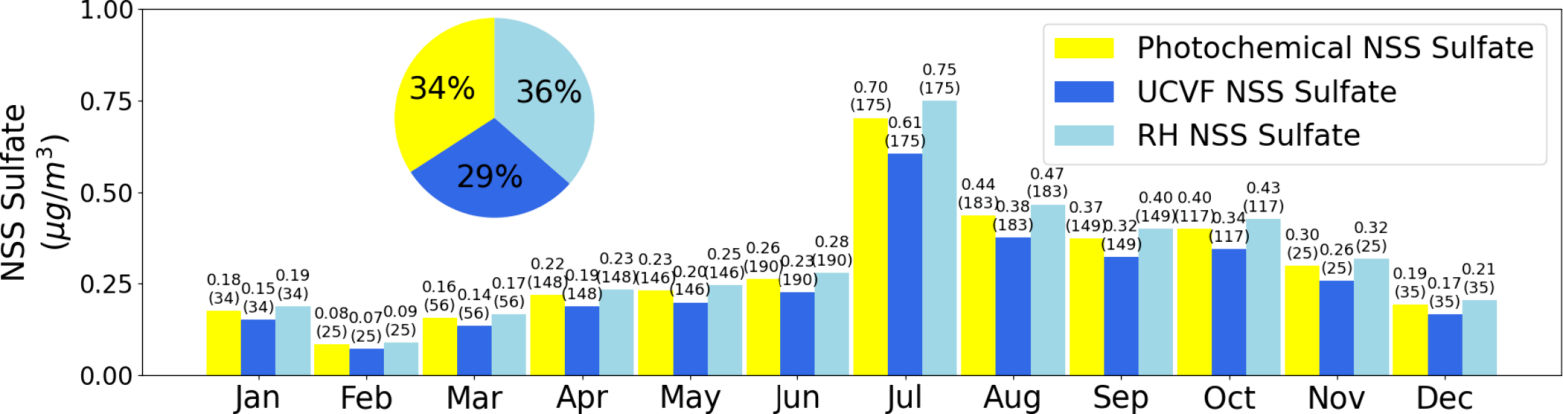
Monthly secondary NSS sulfate mass concentrations per trajectory from source apportionment using MLR analysis comparing regionally emitted NSS sulfate mass contribution from downwelling shortwave radiation (DSW, yellow), upwind cloud vertical fraction (UCVF, blue) and relative humidity (RH, light blue) from the CNW transport cluster. The mean values are shown on top of each bar with and trajectory count in parentheses.

To assess the relative contributions of photochemical and aqueous reactions, MLR using DSW, UCVF, and RH resulted in a Pearson correlation coefficient of 0.46 (Figure 6b), explaining 21% of the variability. Of the three processes, aerosol water uptake (RH) contributed the highest fraction of the regionally emitted sulfate, making up 36% of the measured NSS sulfate mass concentration associated with processing, while photochemical processing based on DSW contributed slightly less at 34% and upwind cloudwater uptake (UCVF) contributed 29% (Figure 11). Incorporating DSW, UCVF and RH with rBC and SST marginally increased the Pearson correlation coefficient of the MLR model of NSS sulfate from 0.50 for sources only to 0.61 for both sources and processes, showing a small improvement in explaining the observed NSS sulfate mass concentration that was formed. The higher fraction of aerosol-water processing is likely explained by the relatively continuous high-RH conditions compared to the intermittency of sunlight and clouds over the region.

## 4 Conclusion

This study used 12 months of measurements of the chemical composition of PM1 at Scripps Pier in La Jolla, California, to evaluate the SO_2_ sources and upwind photochemical and aqueous production of NSS sulfate components. Two source apportionment methods, namely multiple linear regression (MLR) and the tracer method, consistently indicated that the majority of regionally emitted NSS aerosol sulfate originated from marine biogenic emissions (76–80%), with the remaining contributions from regional shipping emissions (20–24%). The 20% contribution from shipping emissions is based on only 26% of the variance in NSS sulfate explained by the rBC and SST included in the MLR method. This 20% shipping contribution is likely an underestimate because of springtime removal of refractory black carbon (rBC) mass concentrations by upwind precipitation. The higher shipping sulfate estimated by the rBC tracer method of 24% is likely an upper bound due to the uncertainties and variability in the ratio of sulfate to rBC in ship emissions.

The influence of upwind cloud processing on secondary aqueous-phase sulfate formation during transport over the ocean was related to the retrieved upwind cloud vertical fraction (UCVF) and the local cloud-mean liquid water content at Scripps Pier. UCVF showed moderate correlations with NSS sulfate mass concentrations in April, May, June, and August (r = 0.32–0.41, *p* < 0.05), suggesting that upwind cloud processing contributes to secondary sulfate formation during these cloudy months. The local cloud-mean LWC did not have a positive significant correlation with NSS sulfate mass in any month. Our results suggest that while cloudwater can enhance the rate of water uptake,^40,44,45^ the cloud vertical fraction may better represent the duration and frequency of cloud events, which in turn influences sulfate formation when water is in excess. Overall NSS sulfate mass was observed to depend on relative humidity (RH) during the winter months, when RH showed considerable variation, unlike the cloudy months that had RH consistently above 80%. MLR analysis explained 21% of the variability related to oxidation processes and supported the attribution of 36% of regional sulfate production to RH-related aerosol water reactions, 34% to DSW-related photochemical reactions, and 29% to UCVF-related cloudwater reactions.

Together these results show the value of long-term surface and satellite aerosol and cloud measurements in coastal areas, providing the ability not only to isolate anthropogenic and biogenic sources but also to characterize the sulfate production mechanism. Such quantification provides essential constraints for improving aerosol process representation in air quality and climate models.

## Supporting Information Available

Additional details on measurements, methods, and results, including supplemental figures and tables to support the main text (PDF)
