# Supplementary material for: Photochemical and Cloud and Aerosol Aqueous Contributions to Regionally-Emitted Shipping and Biogenic Non-Sea-Salt Sulfate Aerosol in Coastal California
Source: ACS EST Air. 2025 Mar 19;2(4):648–64. doi: 10.1021/acsestair.4c00352 (PMC11997993; doi:10.1021/acsestair.4c00352)
Supplement: Supplementary file 1 — ea4c00352_si_001.pdf [file ea4c00352_si_001.pdf]

Photochemical and Cloud and Aerosol Aqueous Contributions to Regionally-Emitted Shipping and Biogenic Non-Sea-Salt Sulfate Aerosol in Coastal California

Nattamon Maneenoi[1], Lynn M. Russell[1*], Sanghee Han[1], Jeramy L. Dedrick[1], Abigail S. Williams[1], Veronica Z. Berta[1], Christian Pelayo[1], Maria A. Zawadowicz[2], Arthur J. Sedlacek III[2], Israel Silber[3], Mandy Thieman[4], David Painemal[4], and Samuel S.P. Shen[1,5]

[1]Scripps Institution of Oceanography, University of California, San Diego, La Jolla, CA 92037, USA.
[2]Brookhaven National Laboratory, Upton, NY 11973, USA.
[3]Pacific Northwest National Laboratory, Richland, WA 99352, USA.
[4]NASA Langley Research Center, Hampton, VA 23681, USA.
[5]San Diego State University, San Diego, CA 92182, USA.

*Email: lmrussell@ucsd.edu

Supporting Information

This supporting information provides additional details on the methodological procedures, results (Figs. S1-4, S6, S8-9, S10-11), comparisons of methods (Figs. S5, S7), and tables that present numerical results (Tables S1-S3, S5) and summarize relevant literature (Tables S4, S7).

Retrieved Ship Speed Over Ground (SOG)

The hourly gridded ship speeds over ground (SOG), excluding ships with a SOG of zero, were averaged to compute the SOG per trajectory. The monthly boxplot of the retrieved SOG is shown in Fig. S8b. SOG does not account for sea state and ocean conditions changing the power required for a given distance traveled, but these were the most representative available metrics for representing ship-to-ship variability in emissions. The 3-hour SOG showed a weak, statistically significant correlation with NSS sulfate concentrations ($r = 0.26$, $p < 0.05$) (Table S2). The increase in NSS sulfate concentrations with SOG aligns with previous findings of engine load and ship speed dependence of the conversion efficiency for fuel sulfur into sulfate (Petzold et al., 2010; Lack et al., 2011). Increased emissions of rBC and particles containing NSS sulfate and nitrate were observed with higher ship speed, which corresponds to reduced fuel efficiency of the

combustion process at higher engine load (Petzold et al., 2008; Lack et al., 2009; Cappa et al., 2014; Betha et al., 2017; Price et al., 2017). However, the 3-hour SOG did not show a significant correlation with rBC (Table S2), likely because lower engine loads result in higher rBC emissions per unit of fuel (Petzold et al., 2010) or a non-linear relationship (Heikkila et al., 2024). Nitrate did not show any significant correlation with SOG (Table S2), likely due to its small fraction in fresh ship-emitted particles (Cappa et al., 2014). The monthly correlations between rBC, NSS sulfate, and nitrate mass concentrations with SOG were not statistically significant.

Figure S1. Density plots of 48 hr back-trajectories in 0.1-degree latitude and longitude bins originating at Scripps Pier, La Jolla, CA from each transport cluster group (a) Coastal NW cluster (64%), (b) LA-LB cluster (18%), (c) Southerly cluster (6%), (d) Easterly cluster (9%), (e) Westerly cluster (3%).

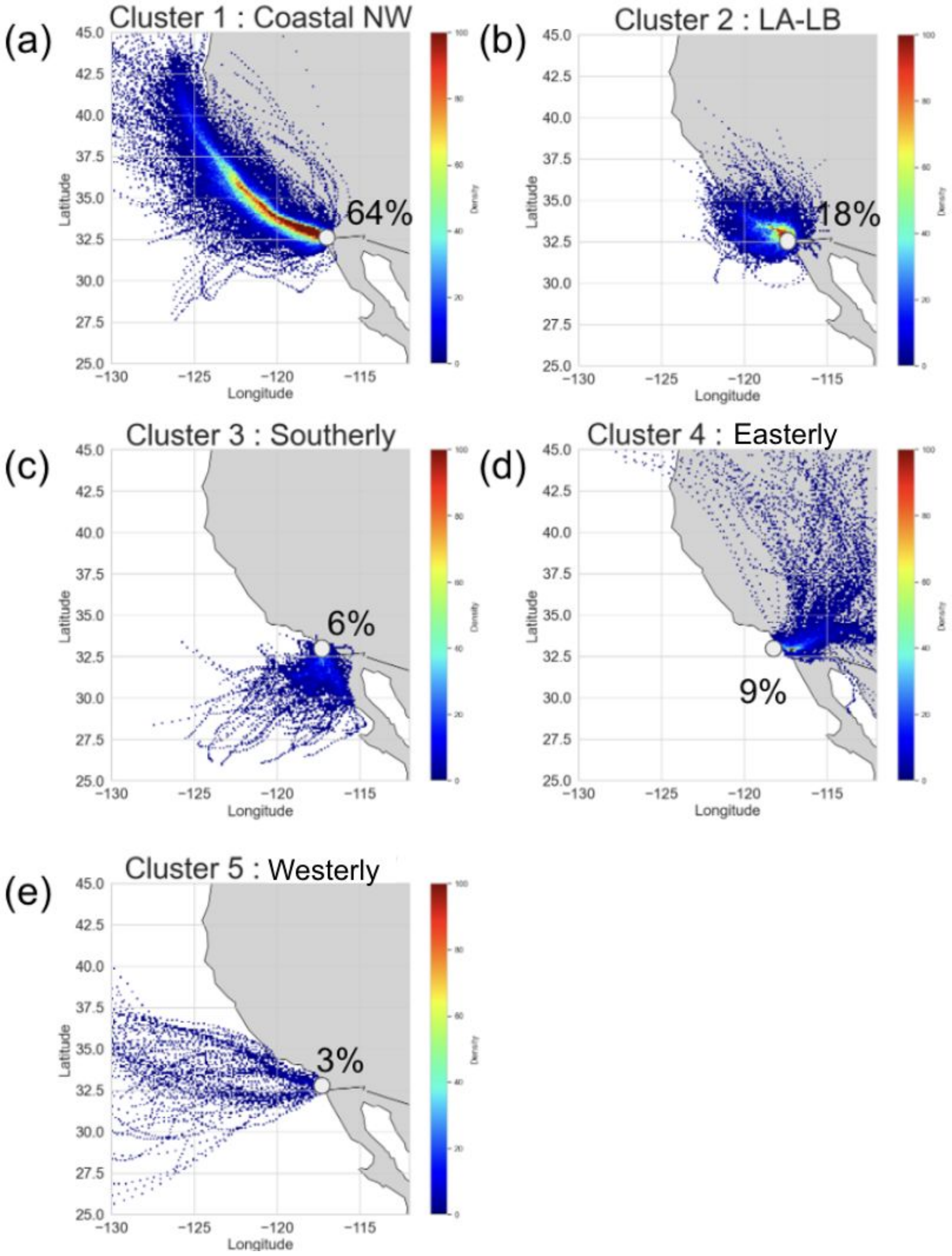

Figure S2. The monthly ship count density for the year 2023, retrieved from the NOAA Automatic Identification System (AIS). The data encompasses passenger, cargo, and tanker vessels and is processed into hourly gridded point densities. The geographical coverage spans from latitudes 32°N to 34.5°N and longitudes 124°W to 116°W, with a resolution of 0.1 degrees for both latitude and longitude bins. The color scale indicates the number of points, representing the density of ship traffic in each grid cell.

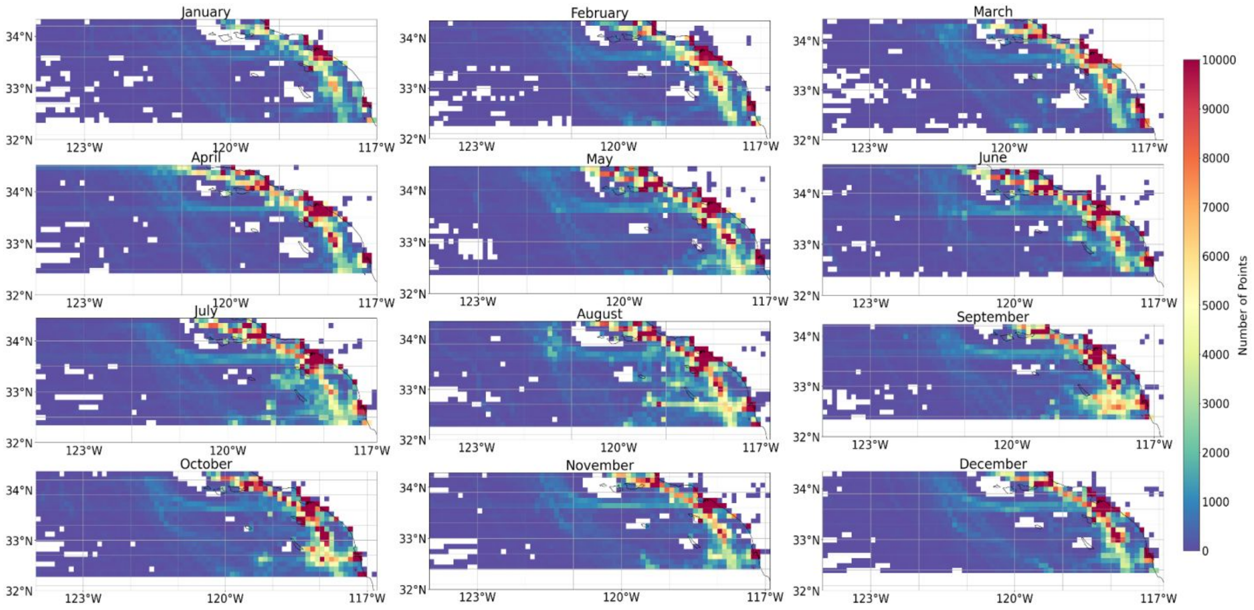

Figure S3. The monthly average Sea Surface Temperature (SST) during the EPCAPE campaign, retrieved from the ERDDAP server of NOAA CoastWatch. The data is derived from the Gap-filled Data Interpolating Empirical Orthogonal Functions (DINEOF) method, providing a high resolution of 5 km. The geographical coverage spans from latitudes 31°N to 36°N and longitudes 129°W to 114°W. The color scale represents the average SST values in each grid cell.

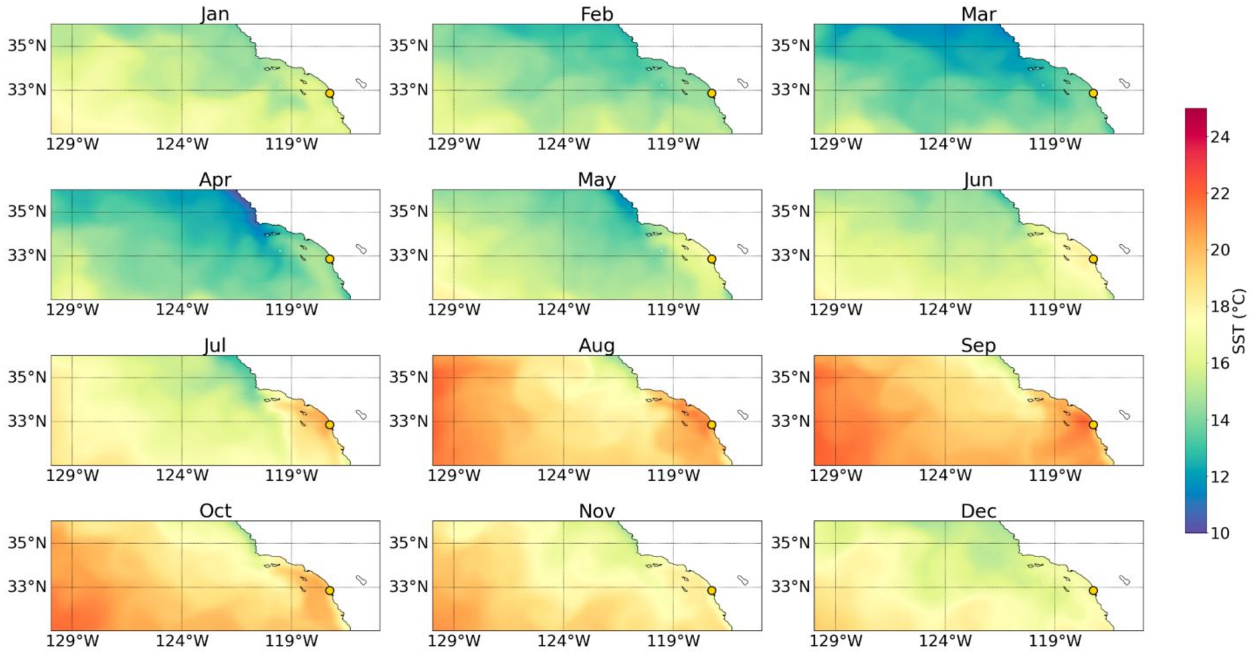

Figure S4. The monthly average Chlorophyll *a* (Chl *a*) during the EPCAPE campaign, retrieved from the ERDDAP server of NOAA CoastWatch. The data is derived from the Gap-filled Data Interpolating Empirical Orthogonal Functions (DINEOF) method, providing a high resolution of 2 km. The geographical coverage spans from latitudes 31°N to 36°N and longitudes 129°W to 114°W. The color scale represents the average SST values in each grid cell.

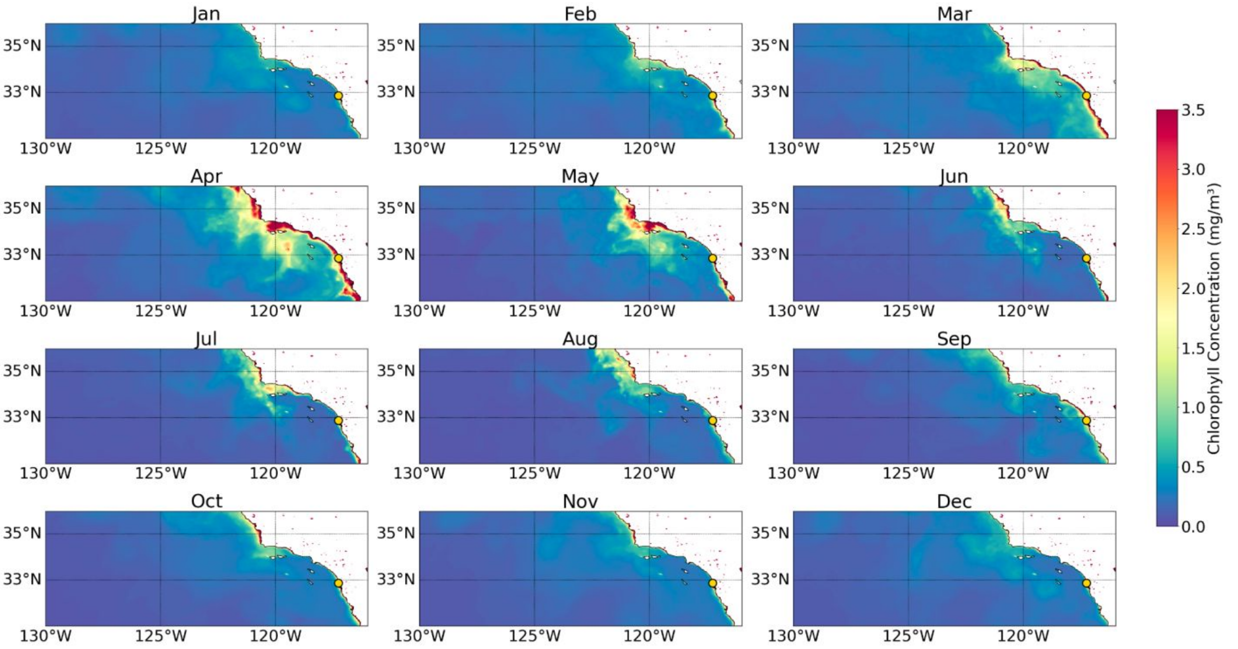

Figure S5. Density scatter plots comparing retrieved cloud and Planetary Boundary Layer Heights (PBLH) at Scripps Pier: (a) SatCORPS satellite-retrieved Cloud Top Height (CTH) versus ARM KAZR CTH measurements reported in ARSCL, (b) SatCORPS satellite-retrieved Cloud Base Height (CBH) versus ARM KAZR CBH measurements, (c) SatCORPS satellite-retrieved CTH versus PBLH derived from radiosonde measurements using the Heffter method, and (d) PBLH derived from radiosonde measurements using the Richardson number method (threshold of 0.25) versus PBLH derived from the Heffter method. Each plot includes a linear regression fit, with the Pearson correlation coefficient and p-value provided in the legend.

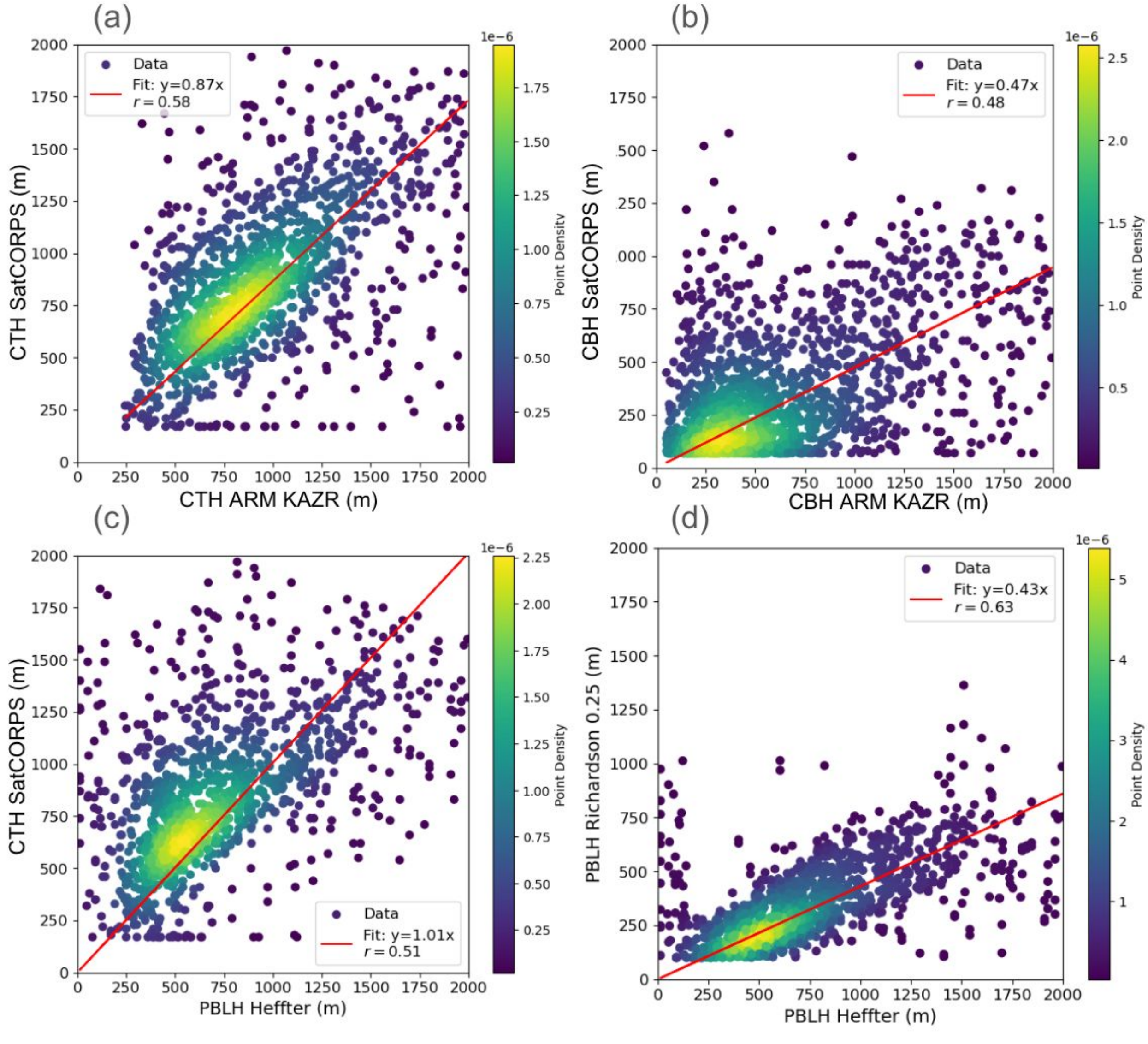

Figure S6. Time series of meteorological conditions during EPCAPE: (a) sea surface temperature (°C, yellow) and ambient air temperature (°C, orange); (b) relative humidity (%, light blue) and precipitation rate (mm/day, navy); (c) 24-hr averaged air mass altitude (maroon), cloud base height (CBH) (blue), cloud top height (CTH) (green) in meters unit; (d) cloud LWP (g/m^2) measured at Scripps Pier (royal blue); (e) local cloud base height (CBH) (blue) and cloud top height (CTH) (green) measured at Scripps Pier in meters unit. (a) and (b) were retrieved and calculated from AOSMET at Scripps Pier, La Jolla, California. (c) was computed by utilizing the Minnis cloud products using VISST algorithm and the ARMTRAJ trajectories and (d) and (e) were retrieved from the ARM ARSCL products processed using the local measurements at Scripps Pier from Feb 15, 2023 - Feb 14, 2024.

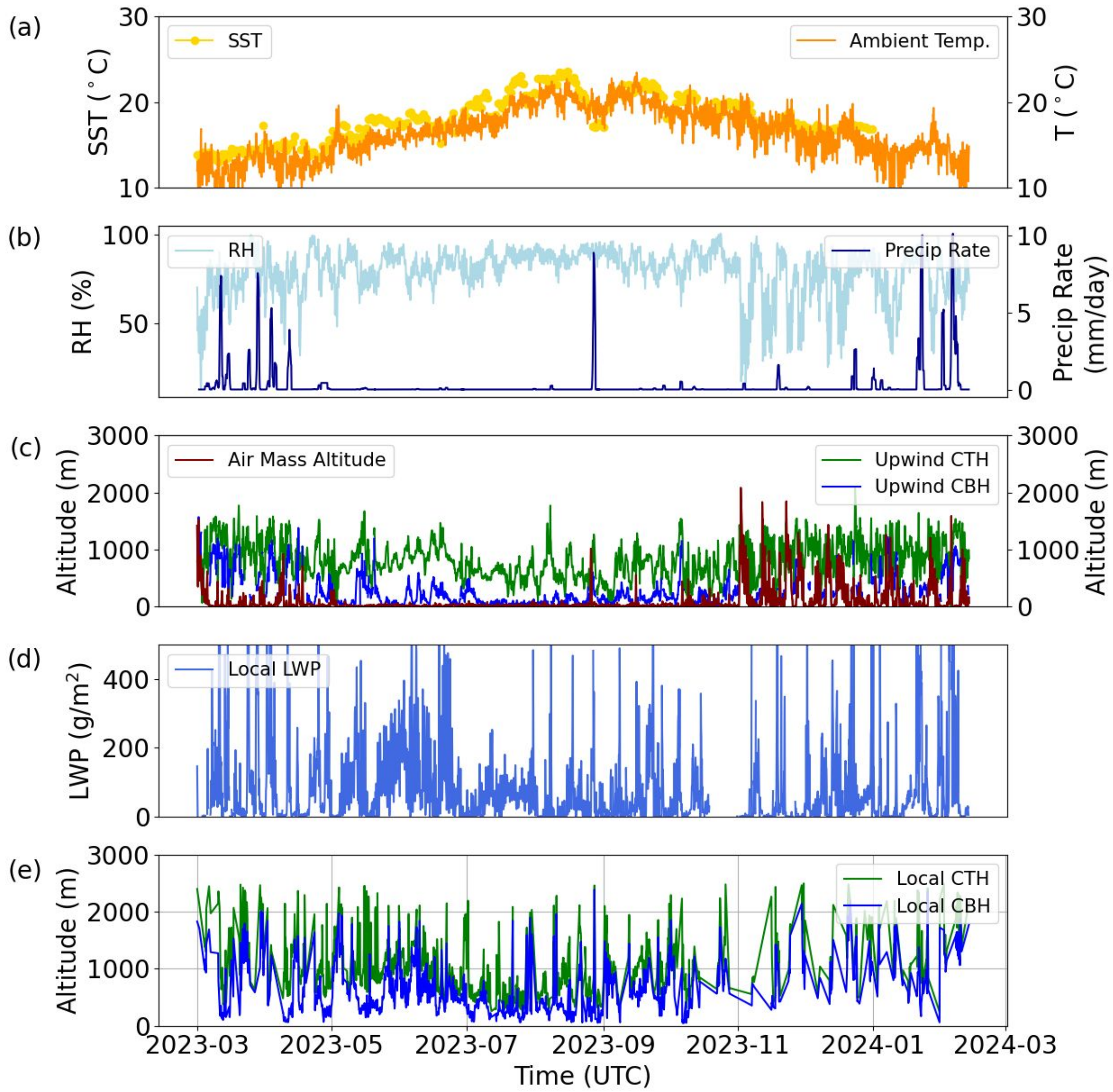

Figure S7. Scatter plot comparing the sea surface temperature (SST) (°C) from real-time measurements at Scripps Pier, provided by the Shore Stations Program during EPCAPE with (a) ambient air temperature (°C) measured from the MET station and with (b) the retrieved SST along trajectory (°C) from NOAA CoastWatch daily data. The plot includes a linear regression fit, with Pearson correlation coefficient and p-value shown in the legend. Data points are colored by month.

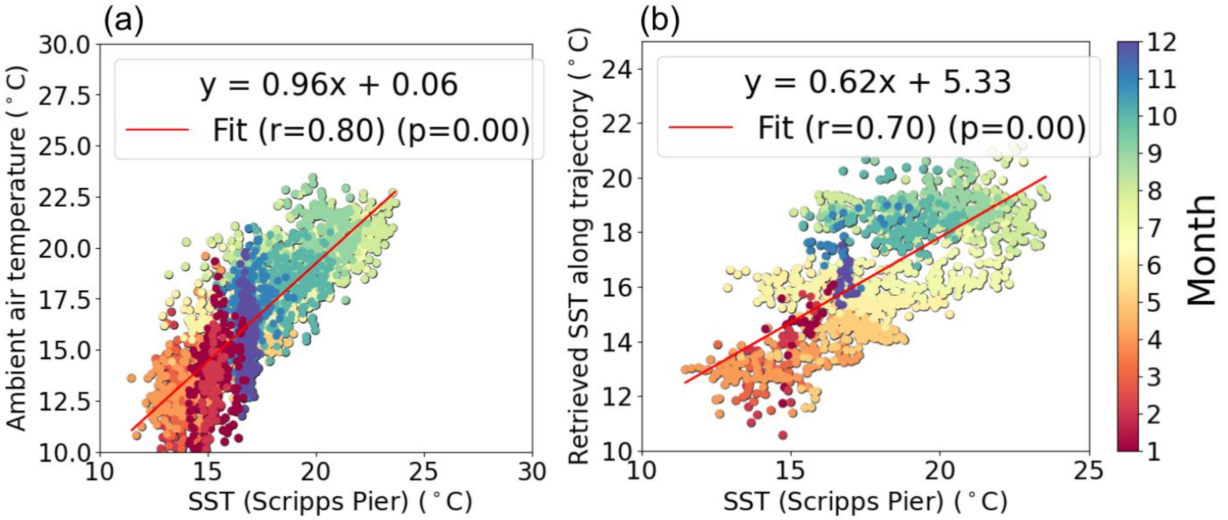

Figure S8. Monthly box plots of (a) the retrieved ship count sum per trajectory and (b) the retrieved average ship speed over ground (SOG) (knots). Monthly medians and sample sizes are denoted as blue text below the box plot for each month. Data was only available for 2023; therefore, January and February data were not present.

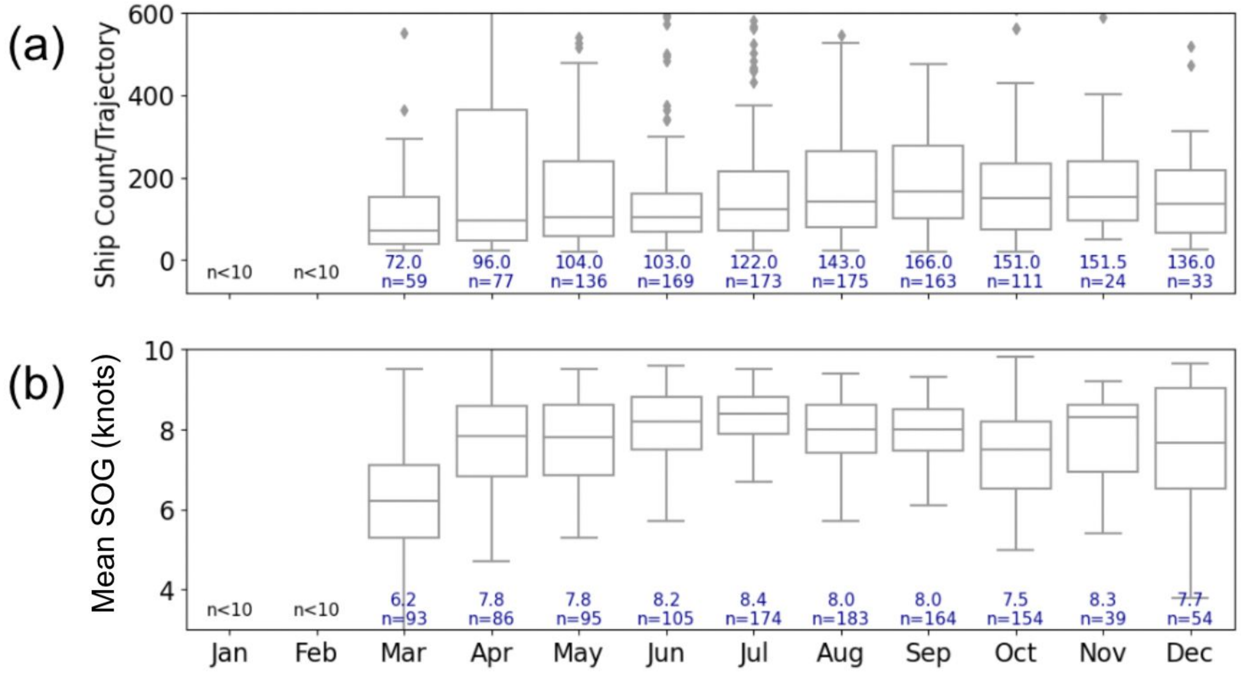

Figure S9. The 240-hr or 10-day back trajectories originating at Scripps Pier, La Jolla, CA showing air mass transport history during the Kīlauea volcanic eruptions in 2023: (a) Jan 5 - Mar 7, (b) June 7-19, and (c) Sept 10-15. The eruption periods were extended by an additional 5 days to allow sufficient time for SO_2 to reach the study site. Note that for the first eruption period, our analysis only considers trajectories that arrived at the site starting from February 15, 2023, which marks the beginning of our campaign.

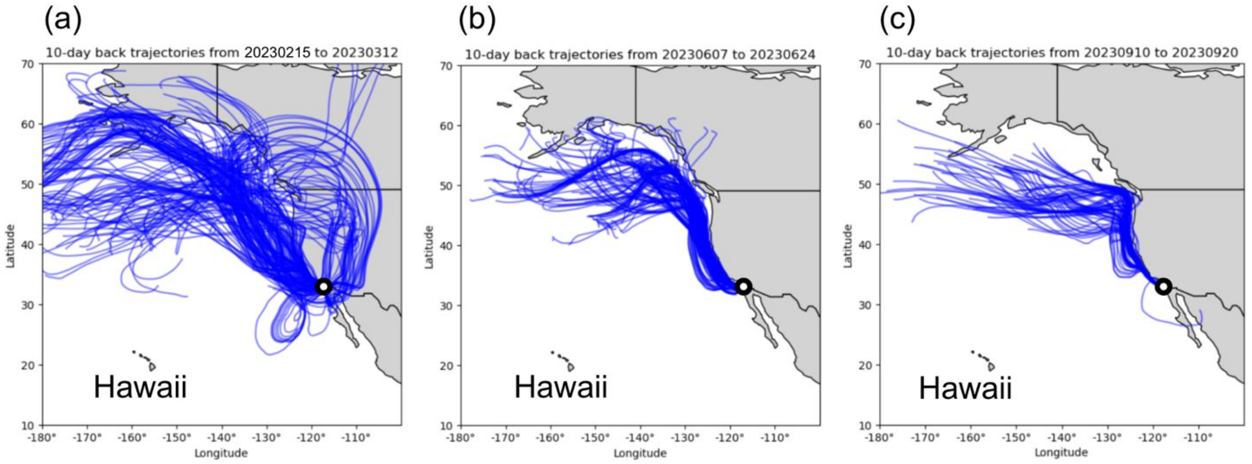

Figure S10. Time series of residuals between the standardized measured NSS sulfate and the standardized predicted NSS sulfate from (a) rBC, (b) source precursors (rBC and SST), (c) process precursors (DSW, UCVF, RH), and (d) both source and process precursors (rBC, SST, DSW, UCVF, RH). The solid gray line represents the residuals for each case, the red dashed line indicates the mean residual, and the blue dashed line shows the mean squared residual, with their values provided in the legend. The three Kīlauea eruption periods (Jan 5 - Mar 7, 2023; June 7-19, 2023; September 10-15, 2023, with 5 additional extended days) are highlighted in yellow in panel (a). For the first period, analysis is only considered from February 15 - March 7, 2023, due to the start of our campaign on February 15. The mean residual values are provided for each eruption period. These eruption periods indicate that NSS sulfate was underpredicted with respect to rBC mass concentrations, suggesting no NSS sulfate enhancement during these volcanic eruptions.

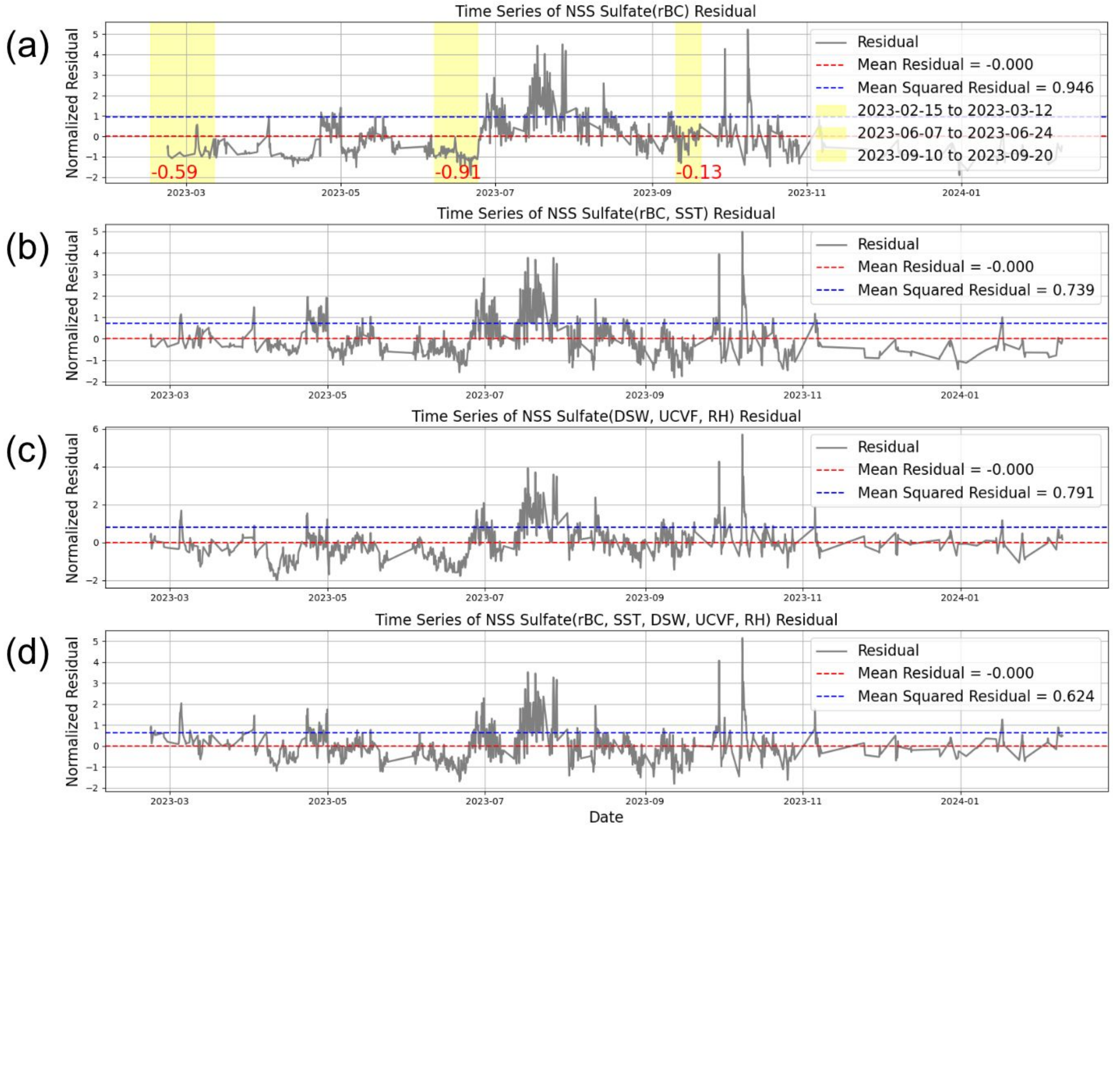

Figure S11. Monthly point-density scatter plots of NSS sulfate mass concentration (μg/m^3) vs. local cloud-mean Liquid Water Content (LWC) (g/m^3). Linear regression lines are fitted where significant correlations are observed ($|r| > 0.2$ and $p < 0.05$), with the corresponding regression equation, Pearson correlation coefficient (r), and p-value indicated on the plots.

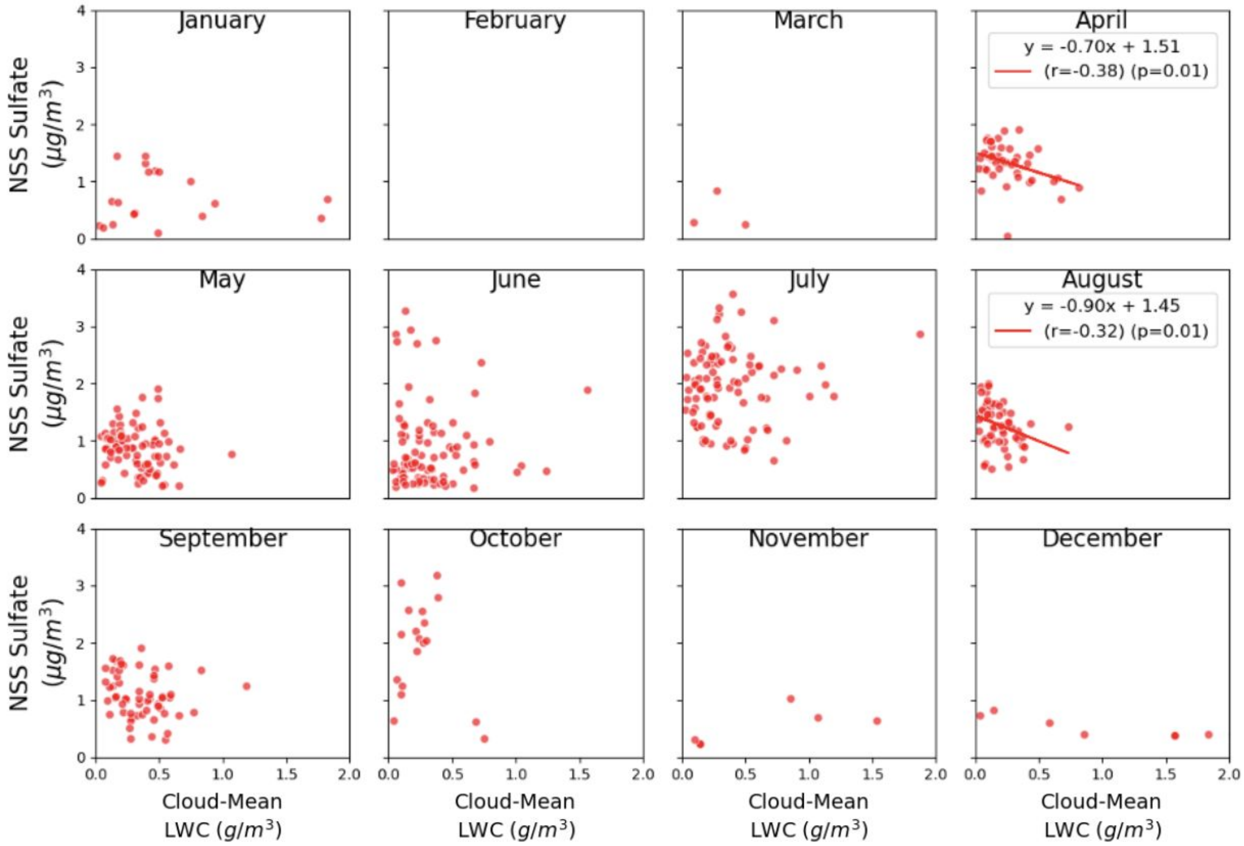

Table S1. Monthly mean and standard deviation of observed meteorological parameters and PM1 aerosol composition, including ACSM non-refractory (NR) species, SP2 rBC, sea salt and dust during EPCAPE at Scripps Pier.

	Jan 2024	Feb*	Mar 2023	Apr 2023	May 2023	Jun 2023	Jul 2023	Aug 2023	Sep 2023	Oct 2023	Nov 2023	Dec 2023	Campaign Avg.
**Ambient air temp (°C)**	14.20 ± 1.80	12.79 ± 1.73	12.90 ± 1.53	13.73 ± 1.57	15.53 ± 0.85	16.89 ± 0.81	19.10 ± 1.48	19.98 ± 1.17	20.06 ± 1.33	18.34 ± 1.27	16.95 ± 1.54	15.69 ± 1.50	16.33 ± 2.87
**RH (%)**	73.71 ± 16.13	69.36 ± 14.43	76.81 ± 12.27	81.16 ± 12.16	80.51 ± 7.63	81.64 ± 6.10	87.79 ± 4.64	88.64 ± 4.08	83.27 ± 5.53	82.95 ± 14.43	66.09 ± 20.48	75.49 ± 14.71	78.8 ± 13.9
**PWD* precipitation rate (mm/hr)**	0.05 ± 0.38	0.15 ± 0.46	0.10 ± 0.35	0.00 ± 0.03	0.00 ± 0.01	0.00 ± 0.01	0.00 ± 0.01	0.04 ± 0.29	0.00 ± 0.01	0.00 ± 0.04	0.01 ± 0.05	0.02 ± 0.17	0.03 ± 0.23
**NR Organics (µg/m^3)**	1.99 ± 1.49 (54%)	1.04 ± 1.32 (42%)	1.14 ± 1.32 (41%)	1.30 ± 1.50 (41%)	0.91 ± 0.77 (38%)	0.92 ± 0.66 (40%)	2.04 ± 0.81 (38%)	2.05 ± 0.99 (47%)	2.28 ± 1.28 (51%)	3.20 ± 2.34 (50%)	2.91 ± 3.28 (51%)	2.95 ± 1.92 (51%)	1.90 ± 1.8 (49%)
**NR Nitrate (µg/m^3)**	0.59 ± 0.89 (12%)	0.19 ± 0.44 (6%)	0.22 ± 0.32 (8%)	0.27 ± 0.39 (8%)	0.17 ± 0.22 (6%)	0.09 ± 0.06 (4%)	0.27 ± 0.23 (5%)	0.22 ± 0.23 (5%)	0.25 ± 0.33 (5%)	0.73 ± 1.10 (9%)	0.56 ± 0.87 (9%)	0.84 ± 1.00 (12%)	0.37 ± 0.7 (9%)
**NR NSS Sulfate (µg/m^3)**	0.28 ± 0.28 (10%)	0.25 ± 0.21 (17%)	0.42 ± 0.33 (24%)	0.56 ± 0.60 (19%)	0.64 ± 0.33 (33%)	0.71 ± 0.64 (30%)	2.00 ± 0.76 (38%)	1.25 ± 0.49 (30%)	1.05 ± 0.51 (26%)	1.12 ± 0.70 (22%)	0.42 ± 0.33 (9%)	0.42 ± 0.31 (8%)	0.78 ± 0.70 (21%)
**NR SS Sulfate (µg/m^3)**	0.02 ± 0.01 (1%)	0.01 ± 0.00 (1%)	0.01 ± 0.00 (1%)	0.01 ± 0.01 (1%)	0.00 ± 0.00 (0%)	0.01 ± 0.00 (0%)	0.01 ± 0.00 (0%)	0.01 ± 0.00 (0%)	0.01 ± 0.00 (0%)	0.01 ± 0.00 (0%)	0.02 ± 0.01 (1%)	0.03 ± 0.02 (1%)	0.01 ± 0.01 (0%)
**NR Ammonium (µg/m^3)**	0.27 ± 0.42 (6%)	0.14 ± 0.23 (8%)	0.30 ± 0.39 (13%)	0.41 ± 0.44 (15%)	0.41 ± 0.51 (17%)	0.32 ± 0.31 (14%)	0.87 ± 0.35 (16%)	0.62 ± 0.28 (14%)	0.53 ± 0.36 (12%)	0.79 ± 0.84 (12%)	0.32 ± 0.44 (5%)	0.41 ± 0.44 (6%)	0.46 ± 0.5 (12%)
**NR NSS Chloride (µg/m^3)**	0.07 ± 0.06 (2%)	0.09 ± 0.11 (6%)	0.04 ± 0.05 (2%)	0.05 ± 0.05 (3%)	0.03 ± 0.03 (2%)	0.03 ± 0.01 (2%)	0.05 ± 0.04 (1%)	0.05 ± 0.03 (1%)	0.06 ± 0.07 (1%)	0.10 ± 0.11 (2%)	0.07 ± 0.07 (2%)	0.12 ± 0.13 (2%)	0.06 ± 0.08 (1%)
**SP2 rBC (ng/m^3)**	45.4 ± 35 (1%)	19.6 ± 22 (0%)	7.4 ± 8 (0%)	16.1 ± 25 (1%)	11.8 ± 15 (1%)	30.0 ± 23 (2%)	40.5 ± 34 (1%)	27.5 ± 24 (1%)	35.7 ± 34 (1%)	50.2 ± 48 (2%)	67.3 ± 50 (1%)	75.2 ± 55 (3%)	0.03 ± 0.04 (1%)
**Sea salt (µg/m^3)**	0.18 ± 0.20 (15%)	0.10 ± 0.08 (17%)	0.07 ± 0.04 (7%)	0.08 ± 0.05 (10%)	0.03 ± 0.02 (3%)	0.05 ± 0.04 (5%)	0.03 ± 0.02 (1%)	0.04 ± 0.02 (1%)	0.04 ± 0.04 (4%)	0.06 ± 0.04 (2%)	0.21 ± 0.30 (11%)	0.35 ± 0.30 (13%)	0.11 ± 0.10 (3%)
**Dust (µg/m^3)**	0.09 ± 0.10 (8%)	0.06 ± 0.07 (10%)	0.05 ± 0.05 (6%)	0.03 ± 0.03 (4%)	0.03 ± 0.01 (3%)	0.05 ± 0.08 (5%)	0.05 ± 0.05 (1%)	0.05 ± 0.09 (1%)	0.04 ± 0.02 (3%)	0.13 ± 0.20 (5%)	0.43 ± 0.80 (13%)	0.30 ± 0.20 (11%)	0.11 ± 0.12 (3%)

Feb*: Feb 15-28, 2023, and Feb 1-14, 2024

PWD precipitation rate*: Precipitation rate measured by the Present Weather Detector (PWD) at Scripps Pier.

Table S2. Summary of linear regression based on data points from the Coastal NW cluster (i.e., 3-hour trajectories). The table includes Y and X variables, the fitted linear equations, Pearson correlation coefficients (r values), and p values.

Y variable	X variable	Fitted linear equation	r value	p value
rBC ($\mu g/m^3$)	Ship count per trajectory	Y = 0.00X + 0.03	0.07	0.01
rBC ($\mu g/m^3$)	Ship Speed per trajectory (SOG) (knots)	Y = 0.00X + 0.01	0.11	0.00
rBC ($\mu g/m^3$)	Local SST (°C)	Y = 0.00X - 0.02	0.23	0.00
Nitrate ($\mu g/m^3$)	Ship count per trajectory	Y = 0.00X + 0.28	0.02	0.57
Nitrate ($\mu g/m^3$)	Ship Speed per trajectory (SOG) (knots)	Y = 0.01X + 0.22	0.02	0.52
NSS Sulfate ($\mu g/m^3$)	rBC ($\mu g/m^3$)	Y = 6.77X + 0.86	0.23	0.00
NSS Sulfate ($\mu g/m^3$)	Ship count per trajectory	Y = 0.00X + 1.00	0.08	0.00
NSS Sulfate ($\mu g/m^3$)	Ship Speed per trajectory (SOG) (knots)	Y = 0.15X – 0.06	0.27	0.00
NSS Sulfate ($\mu g/m^3$)	Nitrate ($\mu g/m^3$)	Y = 0.31x + 0.95	0.21	0.00
NSS Sulfate ($\mu g/m^3$)	Local SST (°C)	Y = 0.15X - 1.65	0.50	0.00
NSS Sulfate ($\mu g/m^3$)	Regional SST (°C)	Y = 0.16X - 1.36	0.40	0.00
NSS Sulfate ($\mu g/m^3$)	Local Chl *a* (mg/m^3)	Y = -0.02X + 1.18	-0.22	0.00
NSS Sulfate ($\mu g/m^3$)	Regional Chl *a* (mg/m^3)	Y = -0.62X + 1.14	-0.28	0.00
NSS Sulfate ($\mu g/m^3$)	Wind speed (m/s)	Y = -0.10X + 1.29	-0.19	0.00
NSS Sulfate ($\mu g/m^3$)	DSW (W/m^2)	Y = 0.00X + 0.7	0.15	0.00
NSS Sulfate ($\mu g/m^3$)	Upwind Cloud Vertical Fraction (UCVF)	Y = 0.98X + 0.44	0.28	0.00
NSS Sulfate ($\mu g/m^3$)	Local Cloud Liquid Water Content (LWC) (g/m^3)	Y = -0.00X + 1.09	-0.02	0.63
NSS Sulfate ($\mu g/m^3$)	RH (%)	Y = 0.03X – 1.64	0.31	0.00

Table S3. Summary of multiple linear regression (MLR) models used to identify sources of sulfate based on the Coastal NW cluster (i.e., 3-hour trajectories), with variables standardized prior to fitting. The table includes the fitted MLR equations, Pearson correlation coefficients (r values), p values, Mean Squared Errors, and the percentage of variability explained by each predictor.

**MLR Model**	**Fitted MLR Equation**	**r value**	**p value**	**MSE**	**% Contribution**
**NSS Sulfate(rBC, SST)**	**Y = 0.12rBC + 0.47SST**	**0.51**	**0.00**	**0.74**	**rBC: 20%**<br>**SST: 80%**
**NSS Sulfate(DSW, UCVF, RH)**	**Y = 0.29DSW + 0.25UCVF + 0.31RH**	**0.46**	**0.00**	**0.79**	**DSW: 34%**<br>**UCVF: 29%**<br>**RH: 36%**
NSS Sulfate(rBC, SST, DSW)	Y = 0.13rBC + 0.45SST + 0.08DSW	0.52	0.00	0.73	rBC: 20%<br>SST: 68%<br>DSW:12%
NSS Sulfate(rBC, SST, UCVF)	Y = 0.13rBC + 0.43SST + 0.21UCVF	0.55	0.00	0.69	rBC: 20%<br>SST: 68%<br>UCVF:12%
NSS Sulfate(rBC, SST, RH)	Y = 0.09rBC + 0.45SST + 0.25RH	0.57	0.00	0.68	rBC: 11%<br>SST: 57%<br>UCVF:32%
**NSS Sulfate(rBC, SST, DSW, UCVF, RH)**	**Y = 0.11rBC + 0.39SST + 0.20DSW + 0.19UCVF + 0.25RH**	**0.61**	**0.00**	**0.62**	**rBC: 10%**<br>**SST: 34%**<br>**DSW: 18%**<br>**UCVF: 17%**<br>**RH: 22%**

Definition for abbreviations shown in the table: rBC : Refractory black carbon ($\mu g/m^3$) measured by SP2 at Scripps Pier, SST : Sea surface temperature (°C), measured at Scripps Pier, DSW : Downwelling Shortwave Radiation (W/m^2), measured at Scripps Pier, UCVF : Upwind Cloud Vertical Fraction, RH : Relative humidity (%)

Table S4. Reported particle sulfate and black carbon measurements from shipping emissions off the California coast and ports as well as other regions.

Reference	Instruments, location, and study period	Sampling description	Sulfate	BC	Sulfate/BC
**California Coast**					
This study	Ambient ACSM and SP2, Scripps Pier, La Jolla, CA Feb 2023 - Feb 2024	Measured during the coastal NW trajectory cluster	0.90 μg/m^3	0.04 μg/m^3	40 ± 80
Ault et al., 2010	Ambient ATOFMS and Aethalometer, Port of Los Angeles, Nov 2007	Fresh ship plume (<45 min age)<br>Diesel fuel<br>Bunker Fuel	 2.3%<br>19.2%	 19.4%<br>2.9%	 0.1<br>6.6
Lack et al., 2011	CalNex Aircraft AMS, SP2, Southern California coast, May 2010	Heavy Fuel Oil at 22 knots<br>S_f: 3.15% (reported), 2.6% (calculated)	2.94 g/kg fuel	0.22 g/kg fuel	13.4
		Marine Diesel Oil at 12 knots<br>S_f: 0.07% (reported), 0.2% (calculated)	0.08 g/kg fuel	0.13 g/kg fuel	0.6
Cappa et al., 2012	CalNex Cruise, HR-AMS, SP2, May-July 2010	Fresh ship plume (<5min)<br>Marine Diesel Oil S_f = 0.1%<br>Low speed: 2.9 knots<br>Medium speed: 6.9 knots<br>High speed: 12 knots	 <br><0.05<br><0.12<br><0.13<br>g/kg fuel	 <br>0.008<br>0.10<br>0.16<br>g/kg fuel	 <br>6.3<br>1.2<br>0.8
Sahu et al., 2012	ARCTAS-CARB Aircraft, California Coast, June 2008	Fossil fuel plume, California<br>Fossil fuel plume, LA regions	18.44%<br>31.0 ± 17 μg/m^3	2.6%<br>1.9 ± 1.0 μg/m^3	7.1<br>16.3
Price et al., 2017	Biofuel cruise, AMS, SP2, California Coast, Sep – Oct 2014 and Sep 2015	HDRD 700 rpm engine speed<br>HDRD 1600 rpm engine speed<br>ULSD 1600 rpm engine speed<br>Polluted marine period	0.7<br>1.2<br>0.23<br>0.67<br>μg/m^3	0.062<br>0.19<br>0.17<br>0.05<br>μg/m^3	11.3<br>6.4<br>1.4<br>13.2
**Other Regions**					
Lack et al., 2009	TexAQS/GoMACCS Cruise, Summer, 2006	Intercept freshly formed ship plume	46%	15%	3.1
Huang et al., 2018	Research Cruise, Atlantic Ocean	Spring 2011<br>(N Atlantic >5°N)<br>(S Atlantic >5°S)<br>(Tropical Atlantic 5°S to 5°N)	 51%<br>57%<br>50%	 4%<br>3%<br>7%	 12.8<br>19<br>7.1
		Fall 2012<br>(N Atlantic >5°N)<br>(S Atlantic >5°S)	 42%<br>47%	 7%<br>3%	 6<br>15.7
Yu et al., 2020	Aircraft measurement, English Channel and North Atlantic Ocean, Sep 2019	S_f > 0.5%<br>S_f < 0.5%	2.58<br>0.16<br>g/kg fuel	0.16<br>0.08<br>g/kg fuel	16.1<br>2
Stavroulas et al., 2021	Ambient Observation, Piraeus, Greece	Winter 2019<br>Summer 2019	2.48<br>3.32<br>μg/m^3	2.43<br>2.45<br>μg/m^3	1.0<br>1.4

Definition for abbreviations shown in the table: S_f : Fuel Sulfur content (% by mass), HDRD : Hydrogenation-Derived Renewable Diesel, ULSD : Ultra-low-sulfur diesel.

Table S5. Monthly correlations (Pearson correlation coefficients (r values) and p values) between pairs of variables in the multiple linear regression model for source (rBC, SST) and process (DSW, UCVF, and RH) variables. Cells are highlighted in light green for months where the correlation is moderate or stronger ($r \geq 0.3$ and $p < 0.05$).

	Jan	Feb	Mar	Apr	May	Jun	Jul	Aug	Sep	Oct	Nov	Dec
rBC vs SST	r = 0.03<br>p = 0.656	r = 0.26<br>p = 0.0	r = 0.14<br>p = 0.033	r = 0.22<br>p = 0.0	r = 0.19<br>p = 0.011	r = 0.02<br>p = 0.751	r = 0.34<br>p = 0.0	r = 0.08<br>p = 0.21	r = -0.33<br>p = 0.0	r = -0.11<br>p = 0.096	r = 0.03<br>p = 0.62	r = 0.01<br>p = 0.819
rBC vs DSW	r = 0.11<br>p = 0.079	r = 0.07<br>p = 0.342	r = 0.06<br>p = 0.367	r = 0.18<br>p = 0.005	r = -0.16<br>p = 0.031	r = -0.17<br>p = 0.009	r = -0.19<br>p = 0.005	r = 0.24<br>p = 0.0	r = 0.0<br>p = 0.99	r = 0.04<br>p = 0.563	r = 0.29<br>p = 0.0	r = 0.03<br>p = 0.609
rBC vs UCVF	r = -0.01<br>p = 0.912	r = 0.11<br>p = 0.106	r = -0.12<br>p = 0.065	r = -0.12<br>p = 0.063	r = 0.14<br>p = 0.062	r = 0.17<br>p = 0.01	r = -0.17<br>p = 0.012	r = 0.0<br>p = 0.993	r = -0.14<br>p = 0.056	r = 0.03<br>p = 0.648	r = -0.08<br>p = 0.229	r = -0.24<br>p = 0.0
rBC vs RH	r = -0.06<br>p = 0.362	r = -0.02<br>p = 0.789	r = -0.18<br>p = 0.006	r = -0.23<br>p = 0.0	r = 0.07<br>p = 0.356	r = -0.04<br>p = 0.545	r = 0.07<br>p = 0.295	r = 0.01<br>p = 0.932	r = 0.12<br>p = 0.097	r = -0.0<br>p = 0.998	r = -0.3<br>p = 0.0	r = -0.1<br>p = 0.127
SST vs DSW	r = 0.18<br>p = 0.003	r = -0.25<br>p = 0.0	r = 0.21<br>p = 0.001	r = 0.08<br>p = 0.241	r = -0.14<br>p = 0.03	r = 0.38<br>p = 0.0	r = -0.09<br>p = 0.14	r = 0.05<br>p = 0.466	r = -0.15<br>p = 0.016	r = -0.06<br>p = 0.321	r = 0.12<br>p = 0.05	r = -0.15<br>p = 0.01
SST vs UCVF	r = -0.18<br>p = 0.003	r = 0.17<br>p = 0.009	r = -0.03<br>p = 0.669	r = 0.2<br>p = 0.002	r = 0.08<br>p = 0.235	r = -0.09<br>p = 0.152	r = -0.39<br>p = 0.0	r = 0.19<br>p = 0.002	r = 0.26<br>p = 0.0	r = 0.09<br>p = 0.18	r = 0.14<br>p = 0.027	r = 0.25<br>p = 0.0
SST vs RH	r = -0.07<br>p = 0.254	r = 0.38<br>p = 0.0	r = -0.16<br>p = 0.01	r = -0.05<br>p = 0.424	r = -0.05<br>p = 0.439	r = -0.26<br>p = 0.0	r = 0.07<br>p = 0.298	r = -0.51<br>p = 0.0	r = -0.35<br>p = 0.0	r = 0.15<br>p = 0.019	r = 0.22<br>p = 0.0	r = 0.37<br>p = 0.0
DSW vs UCVF	r = -0.04<br>p = 0.493	r = 0.16<br>p = 0.015	r = 0.12<br>p = 0.056	r = -0.7<br>p = 0.0	r = -0.82<br>p = 0.0	r = -0.34<br>p = 0.0	r = 0.16<br>p = 0.012	r = 0.01<br>p = 0.915	r = -0.35<br>p = 0.0	r = -0.46<br>p = 0.0	r = 0.12<br>p = 0.057	r = 0.08<br>p = 0.165
UCVF vs RH	r = 0.37<br>p = 0.0	r = 0.07<br>p = 0.265	r = 0.32<br>p = 0.0	r = 0.52<br>p = 0.0	r = 0.74<br>p = 0.0	r = 0.25<br>p = 0.0	r = -0.23<br>p = 0.0	r = 0.01<br>p = 0.86	r = -0.3<br>p = 0.0	r = 0.53<br>p = 0.0	r = 0.54<br>p = 0.0	r = 0.25<br>p = 0.0
RH vs DSW	r = -0.48<br>p = 0.0	r = -0.42<br>p = 0.0	r = -0.52<br>p = 0.0	r = -0.61<br>p = 0.0	r = -0.75<br>p = 0.0	r = -0.31<br>p = 0.0	r = -0.41<br>p = 0.0	r = -0.2<br>p = 0.001	r = 0.03<br>p = 0.647	r = -0.27<br>p = 0.0	r = -0.14<br>p = 0.02	r = -0.17<br>p = 0.005

Table S6. Monthly correlations (Pearson correlation coefficients (r values) and p values) between ACSM organics, nitrate, and NSS sulfate with secondary process precursors (DSW, UCVF, and RH). Cells are highlighted in light green for months where the correlation is moderate or stronger ($r \geq 0.3$ and $p < 0.05$).

	Jan	Feb	Mar	Apr	May	Jun	Jul	Aug	Sep	Oct	Nov	Dec
Org vs DWS	r = 0.14<br>p = 0.026	r = 0.27<br>p = 0.0	r = 0.19<br>p = 0.004	r = 0.2<br>p = 0.002	r = -0.54<br>p = 0.0	r = 0.2<br>p = 0.002	r = -0.3<br>p = 0.0	r = 0.2<br>p = 0.002	r = -0.13<br>p = 0.082	r = -0.17<br>p = 0.011	r = 0.41<br>p = 0.0	r = 0.11<br>p = 0.077
Org vs UCVF	r = 0.09<br>p = 0.175	r = -0.04<br>p = 0.614	r = 0.03<br>p = 0.67	r = -0.01<br>p = 0.916	r = 0.45<br>p = 0.0	r = 0.28<br>p = 0.0	r = 0.06<br>p = 0.334	r = 0.22<br>p = 0.0	r = 0.22<br>p = 0.002	r = 0.15<br>p = 0.02	r = -0.05<br>p = 0.535	r = -0.2<br>p = 0.001
Org vs RH	r = 0.12<br>p = 0.075	r = -0.19<br>p = 0.011	r = -0.1<br>p = 0.159	r = -0.18<br>p = 0.007	r = 0.5<br>p = 0.0	r = -0.11<br>p = 0.094	r = 0.23<br>p = 0.0	r = 0.18<br>p = 0.004	r = 0.08<br>p = 0.288	r = 0.33<br>p = 0.0	r = -0.1<br>p = 0.163	r = 0.16<br>p = 0.013
Nitrate vs DSW	r = -0.05<br>p = 0.465	r = 0.11<br>p = 0.129	r = 0.11<br>p = 0.112	r = 0.05<br>p = 0.428	r = -0.41<br>p = 0.0	r = 0.17<br>p = 0.009	r = -0.34<br>p = 0.0	r = 0.04<br>p = 0.553	r = -0.0<br>p = 0.958	r = -0.24<br>p = 0.0	r = 0.22<br>p = 0.002	r = 0.04<br>p = 0.545
Nitrate vs UCVF	r = 0.18<br>p = 0.005	r = 0.09<br>p = 0.25	r = 0.07<br>p = 0.334	r = 0.17<br>p = 0.011	r = 0.37<br>p = 0.0	r = 0.3<br>p = 0.0	r = 0.1<br>p = 0.135	r = 0.37<br>p = 0.0	r = -0.1<br>p = 0.168	r = 0.24<br>p = 0.0	r = -0.02<br>p = 0.793	r = -0.25<br>p = 0.0
Nitrate vs RH	r = 0.27<br>p = 0.0	r = 0.02<br>p = 0.751	r = -0.06<br>p = 0.391	r = -0.04<br>p = 0.501	r = 0.41<br>p = 0.0	r = -0.06<br>p = 0.34	r = 0.1<br>p = 0.128	r = 0.1<br>p = 0.117	r = 0.21<br>p = 0.004	r = 0.31<br>p = 0.0	r = 0.07<br>p = 0.356	r = 0.22<br>p = 0.0
Sulfate vs DSW	r = -0.38<br>p = 0.03	r = -0.16<br>p = 0.43	r = -0.04<br>p = 0.77	r = 0.04<br>p = 0.61	r = -0.35<br>p = 0.00	r = 0.37<br>p = 0.00	r = -0.09<br>p = 0.20	r = -0.23<br>p = 0.00	r = -0.11<br>p = 0.18	r = -0.13<br>p = 0.18	r = 0.71<br>p = 0.00	r = 0.54<br>p = 0.00
Sulfate vs UCVF	r = 0.29<br>p = 0.10	r = -0.24<br>p = 0.25	r = 0.33<br>p = 0.01	r = 0.35<br>p = 0.00	r = 0.33<br>p = 0.00	r = 0.32<br>p = 0.00	r = -0.33<br>p = 0.00	r = 0.41<br>p = 0.00	r = 0.23<br>p = 0.00	r = 0.08<br>p = 0.36	r = 0.19<br>p = 0.36	r = 0.06<br>p = 0.74
Sulfate vs RH	r = 0.29<br>p = 0.10	r = 0.45<br>p = 0.02	r = -0.18<br>p = 0.17	r = 0.14<br>p = 0.08	r = 0.45<br>p = 0.00	r = -0.14<br>p = 0.05	r = 0.30<br>p = 0.00	r = 0.12<br>p = 0.11	r = 0.07<br>p = 0.41	r = 0.42<br>p = 0.00	r = 0.12<br>p = 0.57	r = 0.34<br>p = 0.04

Table S7. Related literature on secondary aqueous phase sulfate formation due to aqueous processing by cloud water and aerosol water.

Reference and location	Sampling Period	% Sulfate mass increase	Identification of Aqueous Processing	Ammonium/Sulfate Ratio
**By cloud water associated with LWC**				
This study, La Jolla, CA	Feb 2023 - Feb 2024 Cloud-mean LWC 0.33 ± 0.3 g/m^3 20 ± 2°C 79 ± 14% RH	~50-260% (upwind clouds)	Percent increase correlated to the average upwind cloud vertical fraction (UCVF) in the last 24 hours along trajectory over the ocean.	0.30-0.68 (monthly median)
Ge et al., 2012 Fresno, CA	Jan 2010 9.8°C, 85% RH	~100%	3 fog/cloud periods with LWC > 0.02 g/m^3 and RH > 97%; sulfate mass of 0.38 ± 0.43 increased to 0.76 ± 0.28 during fog/cloud periods.	2.38 (fog period) 3.03 (non-fog period) 2.45 (entire period)
Gilardorni et al., 2014 Po Valley, Italy	Nov 2011 3°C, ~100% RH	~ -61% (fog scavenging)	14 fog periods identified by LWC >0.08 g/m^3	1.67
Harris et al., 2014 Thüringer Wald, Germany	Sep-Oct 2010 LWC 0.1-0.4 g/m^3 6-8°C	~ 10-40% (Night) ~ 20-200% (Day)	3 cloud events with LWC > 0.1 g/m^3 and no precipitation.	-
Ervens et al., 2018	Model simulation	~210% (Marine) ~120% (Urban) ~190% (Biogenic) ~220% (Background)	Parcel model simulation for air-mass-specific aerosol and gas-phase compositions, LWC >0.01 g/m^3, cloud persisted 40 min/hr.	-
**By aerosol water associated with RH**				
This study La Jolla, CA	Winter 2023 (Oct-Dec 2023 and Jan 2024) 16 ± 2°C 74 ± 18% RH	~3-6% sulfate mass /%RH	Sulfate increase of 0.01-0.05 $\mu g/m^3$/%RH	0.30-0.68 (monthly median)
Sun et al., 2013 Beijing, China	Winter 2011 -8 to 12°C	~3% sulfate mass /%RH	Sulfate increase of 0.6 $\mu g/m^3$/%RH	1.03 (RH<50%) 0.4 (RH>50%)
Hu et al., 2016 Beijing, China	Winter 2010 2.7 ± 4 °C	~5% sulfate mass /%RH	8.7 ± 10.7 $\mu g/m^3$ sulfate at 30 ± 20% RH 26.4 ± 7 $\mu g/m^3$ sulfate at >60% RH	0.89 (Average) 0.55 (RH>60%)
Wu et al., 2018 Nanjing, China	Winter 2015 8.5 °C (0-21°C)	~4% sulfate mass /%RH	7.5 $\mu g/m^3$ sulfate at 60% RH 14 $\mu g/m^3$ sulfate at 80% RH	0.73 (Average)
Wang et al., 2020 Beijing, China	Winter 2016	~9% sulfate mass /%RH	10 $\mu g/m^3$ sulfate at 40-75% RH 40 $\mu g/m^3$ sulfate at >75% RH	-
